# Major volcanic eruptions episodically disrupted the continuity of polar warming through aerosol forcing

**DOI:** 10.1038/s44407-026-00111-3

**Published:** 2026-11-01

**Authors:** Basudev Swain, Scott Osprey, Roy G. Grainger, Rui Song, Marco Vountas

**Affiliations:** 1 National Centre for Earth Observation, Atmospheric, Oceanic and Planetary Physics, University of Oxford, Oxford, UK; 2 Atmospheric, Oceanic and Planetary Physics, University of Oxford, Oxford, UK; 3 Institute of Environmental Physics, University of Bremen, Bremen, Germany

**Keywords:** Climate sciences, Environmental sciences, Natural hazards, Solid Earth sciences

## Abstract

The polar regions are warming rapidly, exceeding the global average due to greenhouse gas emissions from human activities. Using CMIP6 simulations, HadCRUT5 data, and MERRA-2 aerosol records, this study shows that major 20th-century volcanic eruptions, Agung (1963), El Chichón (1982), and Pinatubo (1991) temporarily offset polar warming through sulfate-induced cooling. The Agung eruption suppressed Arctic surface air temperature anomalies for approximately 4–5 years (1964–1968), El Chichón for a similar duration (1983–1987), and Pinatubo for about 4–5 years (1992–1996), demonstrating a consistent multi-year cooling response following each event. Without these eruptions, Arctic surface air temperature anomalies would have increased by up to 0.5 °C, whereas volcanic activity caused short-term cooling ranging from −0.07 °C to −0.4 °C. Arctic minimum temperatures (${T}_{\min }$) are rising faster than maximum temperatures (${T}_{\max }$), indicating asymmetric warming. In contrast, Antarctic responses were weaker, moderated by oceanic and atmospheric dynamics. Collectively, these eruptions produced episodic multi-year cooling that temporarily interrupted the ongoing Arctic warming trajectory, with warming resuming as volcanic aerosol forcing diminished. Spatial patterns reveal pronounced warming in the central Arctic, Greenland, and Siberia in the absence of volcanic forcing, highlighting the role of eruptions in modulating polar warming and providing insights relevant to future geoengineering considerations.

## Introduction

The polar regions, including the Arctic and Antarctica, are among the most climatically sensitive and rapidly changing areas on Earth^1,2^. Observations and climate model projections consistently show that these regions are warming at accelerated rates, though the pace and extent of warming differ significantly between them^1,2^. The Arctic is under the influence of warming at three to four times the global mean^2,3^. The Arctic warming is driven primarily by human-induced greenhouse gas emissions and amplified through feedbacks such as sea ice loss, changes in cloud cover, and surface albedo alterations^3–9^. In contrast, Antarctic warming is generally influenced by the large thermal inertia of its ice sheet, the buffering effects of the Southern Ocean, and complex interactions between natural variability and anthropogenic forcing^10–12^. Understanding these regional differences is essential for projecting global climate impacts, including sea level rise, shifts in ocean circulation, and disruptions to polar ecosystems.

While the influence of long-term anthropogenic forcing, including greenhouse gases and anthropogenic aerosols, on polar warming is well documented^9,13–15^, the climatic influence of explosive volcanic eruptions has also been extensively investigated. Large eruptions can produce substantial short-term global and regional cooling, and volcanic forcing has contributed importantly to climate variability over centennial to millennial timescales^16–18^. The magnitude, duration, and spatial distribution of the temperature response depend on eruption characteristics, aerosol loading and transport, atmospheric circulation, and regional climate feedbacks. However, the extent to which these episodic volcanic perturbations modulate polar warming, particularly the contrasting responses of the Arctic and Antarctic and the differential responses of annual-mean SAT, (${T}_{\min }$), and (${T}_{\max }$), remains less well characterized. Here, we address this aspect using complementary CMIP6 all-forcing, volcanic-only, and no-volcano counterfactual simulations.

Notable stratospheric eruptions, such as Krakatoa (1883), Santa María (1902), Novarupta (1912), Mount Agung (1963), Volcán de Fuego (1974), El Chichón (1982), and Mount Pinatubo (1991), injected substantial sulphur dioxide (SO_2_) into the stratosphere. This SO_2_ eventually forms sulphuric acid aerosols, which modify the Earth’s radiation balance^18–26^. Sulfur dioxide injected into the stratosphere is oxidized to form sulfuric acid (H_2_SO_4_), which condenses with water vapor to produce liquid aerosol droplets^18,22,23^. In climate models, these droplets are typically represented as “sulfate aerosols,” referring to the bulk aerosol mass used in radiative calculations, whereas “sulfuric acid aerosols” describes their underlying chemical composition^18,22,23^. In addition to large volcanic eruptions, smaller-scale aerosol perturbations provide observational analogues of radiative forcing impacts. Ship-track cloud modifications and the recent International Maritime Organization (IMO) 2020 sulfur regulation, which substantially reduced marine fuel sulfur content, have produced detectable regional aerosol reductions and associated radiative adjustments^27,28^. Studies suggest that rapid decreases in sulfate emissions from shipping may have contributed to short-term regional warming trends by reducing aerosol cooling effects^29,30^. Although these perturbations are far smaller than volcanic injections, they illustrate the climatic sensitivity to changes in sulfate aerosol loading and provide real-world constraints on aerosol-radiation-cloud interactions.

Unlike most research focusing primarily on total temperature changes due to various historical anthropogenic forcings^2,3,31–36^, this analysis includes total surface air temperature (SAT), minimum surface air temperature (${T}_{\min }$), and maximum surface air temperature (${T}_{\max }$), variations over polar regions due to volcanic eruptions. The values of ${T}_{\min }$ and ${T}_{\max }$ determine the occurrence and magnitude of cold extremes, heatwaves, cryospheric processes such as snow and ice melt, and critical ecological thresholds by using daily datasets^37,38^.

This study investigates the response of SAT, ${T}_{\min }$, and ${T}_{\max }$ to all the historical volcanic events from 1850 to 2015 across the Arctic and Antarctic using multi-model simulations from the Coupled Model Intercomparison Project Phase 6 (CMIP6)^39^, supplemented by SAT observational data from HadCRUT5^40^ and aerosol optical depth (AOD) from the modern-era retrospective analysis for research and applications, version 2 (MERRA-2) reanalysis^41^. The (${T}_{\min }$) and (${T}_{\max }$) temperatures correspond to the annual means of daily minimum and daily maximum near-surface air temperatures from CMIP6 outputs. These metrics therefore represent climatological averages of daily extrema rather than single-day or single-year absolute extremes. By combining these datasets, this study examines the climatic impacts of volcanic forcing and its association with aerosol loading in the polar atmosphere.

It is shown that major volcanic eruptions temporarily interrupted the ongoing Arctic warming trajectory through episodic multi-year cooling, with warming resuming as volcanic aerosol forcing diminished. Notably, ${T}_{\min }$ shows a more rapid increase than ${T}_{\max }$, revealing an asymmetric warming pattern. These results highlight the role of volcanic aerosols in modulating polar climate variability and underscore the importance of incorporating large volcanic events, as natural forcings, in future polar climate attribution and projection efforts.

## Results

### Temporal evolution of volcanic eruption climatic influence on polar warming

**Fig. 1:**
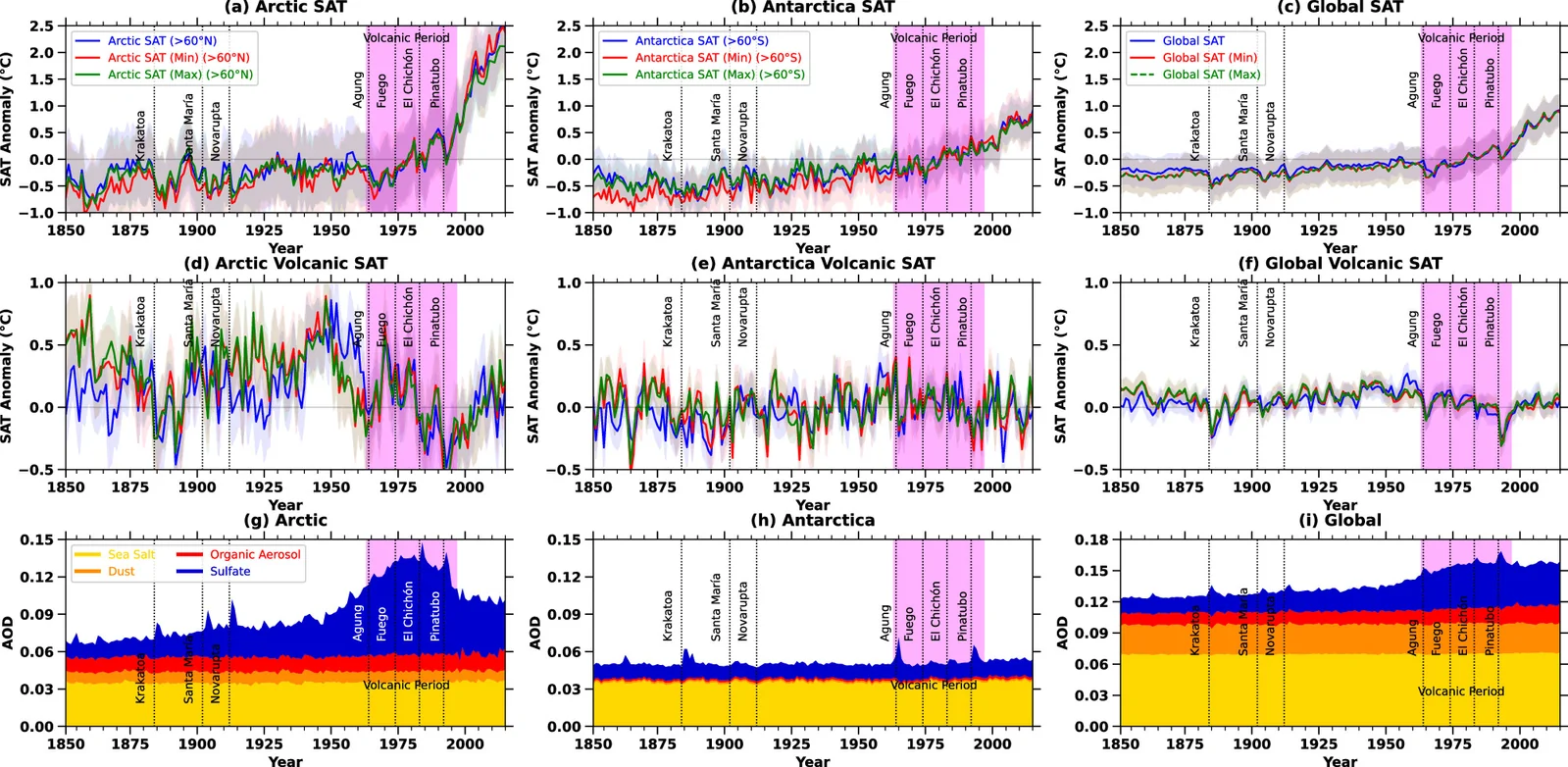
Temporal evolution of SAT anomalies and aerosol optical depth (AOD) over the Arctic, Antarctica, and global domains. **a**–**c** Annual anomalies of total surface air temperature (SAT), minimum temperature (${T}_{\min }$), and maximum temperature (${T}_{\max }$) from CMIP6 historical simulations over the Arctic (>60^∘^N), Antarctica (>60^∘^S), and the global mean, respectively. Anomalies are computed relative to the volcanically influenced baseline period (1964–1996). The shaded purple region indicates the volcanically active period associated with the Agung, El Chichón, and Pinatubo eruptions. **d**–**f** Same as **a**–**c**, but for volcanic-only forcing simulations (hist-volc). **g**–**i** Temporal evolution of total AOD and its components (sulfate, sea salt, dust, and organic aerosol) over the Arctic, Antarctica, and global domains, respectively, derived from the CMIP6 multi-model mean. The vertical dashed lines indicate the timing of major volcanic eruptions, including Krakatoa, Santa Maria, Novarupta, Agung, Fuego, El Chichón, and Pinatubo. These panels illustrate the temporal changes in enhanced sulfate aerosol loading and suppressed SAT anomalies, particularly over the Arctic.

The temporal evolution of total surface air temperature (SAT), minimum temperature (${T}_{\min }$), maximum temperature (${T}_{\max }$), and AOD from 1850 to 2014 over the Arctic, Antarctica, and global domains demonstrates the strong influence of volcanic eruptions on polar climate variability (Fig. 1). The analysis uses CMIP6 multi-model mean (MMM) simulations together with HadCRUT5 SAT observations and MERRA-2 aerosol reanalysis (Table S1, and Figs. S1a, b, and S2). SAT anomalies are calculated relative to the volcanically influenced baseline period (1964-1996), which includes the major eruptions of Agung (1963), El Chichón (1982), and Pinatubo (1991). This baseline provides a consistent framework for comparing multiple eruption events within a common forcing regime. The shaded purple region in Fig. 1 denotes this volcanically active period.

The historical all-forcing simulations, which include both anthropogenic and natural forcings, show a clear warming emerging after the mid-1970s across all regions, with the Arctic displaying the strongest warming (Fig. 1a–c). Arctic SAT anomalies exceed 2 °C by the early 21st century, substantially larger than the global mean response, consistent with previous studies of the Arctic warming^3,8^. The Arctic ${T}_{\min }$ reaches anomalies approaching 2.5 °C, compared to 2.0 °C for ${T}_{\max }$, indicating a stronger warming response in minimum temperatures (Fig. 1a). This enhanced sensitivity of ${T}_{\min }$ reflects the influence of stable boundary-layer conditions, longwave radiative feedbacks, and sea-ice related surface energy changes^4^. Although a stronger response in ${T}_{\min }$ compared to ${T}_{\max }$ may appear counterintuitive under reduced shortwave forcing, several mechanisms explain this behavior^4^. Volcanic sulfate aerosols reduce incoming solar radiation during daytime, but they also modify longwave fluxes and atmospheric stability. Under stable nocturnal boundary-layer conditions, weakened turbulent mixing enhances surface radiative cooling, leading to disproportionately larger reductions in ${T}_{\min }$^42,43^. Aerosol-induced perturbations to surface energy balance and boundary-layer structure have been shown to influence the diurnal temperature range, particularly through enhanced nighttime sensitivity^44,45^. In the Arctic, additional warming arises from sea-ice and surface energy feedbacks that strongly modulate nighttime temperature variability^4,8^. These combined radiative and dynamical processes help explain why ${T}_{\min }$ exhibits a larger response than ${T}_{\max }$ following major eruptions, leading to stronger warming of the coldest extremes^4^.

In contrast, Antarctic warming remains comparatively moderate and closely follows the global mean response (Fig. 1b). This weaker response reflects the combined effects of Southern Ocean heat uptake, the thermal inertia of the Antarctic ice sheet, high elevation of the Antarctic Plateau, strong surface-based inversions, and atmospheric isolation associated with the circumpolar vortex^11,46^. These factors limit vertical mixing and horizontal transport of heat anomalies, thereby reducing the sensitivity of the Antarctic interior to short-term radiative perturbations. Negative anomalies in SAT, ${T}_{\min }$, and ${T}_{\max }$ closely coincide with major volcanic eruptions, particularly over the Arctic, indicating strong sensitivity to volcanic aerosol forcing. The Arctic exhibits cooling responses in all three temperature variables, reflecting its greater sensitivity to volcanic sulfate aerosol forcing. Immediately after the volcanically active period, Arctic warming accelerates rapidly, whereas Antarctica more closely follows the global trend, suggesting a rebound of greenhouse gas-driven warming once volcanic forcing declines. Enhanced humidity-driven warming over newly ice-free regions further contributes to the Arctic warming. Sea-ice loss increases surface evaporation and atmospheric moisture availability, increasing downward longwave radiation at the surface and strengthening surface warming, particularly during autumn and winter when longwave fluxes dominate the surface energy budget^4,8^. Although volcanic forcing temporarily suppresses warming, these humidity-driven feedbacks resume once aerosol loading declines.

The volcanic-only simulations isolate the climatic response to volcanic forcing more clearly (Fig. 1d–f). Distinct cooling signals are visible following the eruptions of Krakatoa (1883), Santa María (1902), Novarupta (1912), Agung (1963), Fuego (1974), El Chichón (1982), and Pinatubo (1991). The Arctic again exhibits the strongest cooling response, particularly in ${T}_{\min }$ relative to ${T}_{\max }$, indicating that volcanic aerosols disproportionately suppress minimum temperatures (Fig. 1d). This differential response can be understood mechanistically. Volcanic aerosols reduce daytime shortwave radiation, moderating ${T}_{\max }$, whereas nighttime temperatures are governed primarily by longwave radiation and boundary-layer stability. Under stable Arctic conditions, weakened turbulent mixing enhances radiative cooling, leading to stronger reductions in ${T}_{\min }$^42,43^. Cloud adjustments likely reflect a combination of mechanisms, including radiative scattering, circulation changes, and aerosol-cloud microphysical interactions^21,47,48^. These combined radiative, dynamical, and microphysical processes amplify nighttime cooling more effectively than daytime cooling, producing larger anomalies in ${T}_{\min }$ relative to ${T}_{\max }$. Antarctic volcanic responses remain weaker and more spatially uniform, reflecting the comparatively lower sensitivity of Antarctic surface temperatures to short-term volcanic radiative forcing.

Figure 1g–i shows the temporal evolution of total AOD and its available aerosol components over the Arctic, Antarctica, and global domains. These AOD fields are not volcanic-only diagnostics; rather, they represent the available CMIP6 total aerosol output. Nevertheless, pronounced increases in sulfate AOD coincide with the timing of major volcanic eruptions and are used here to provide physical context for the eruption-related temperature responses. Comparisons with MERRA-2 reanalysis (Fig. S3a–d) show that CMIP6 captures the timing and overall temporal evolution of aerosol peaks but tends to underestimate their magnitude, particularly for strong Arctic and global eruptions. Arctic aerosol loading remains systematically higher than Antarctic loading, reflecting hemispheric asymmetry in aerosol transport and deposition. During approximately 1950–1995, Arctic aerosol loading was strongly influenced by anthropogenic sulfate emissions from major Northern Hemisphere source regions, including North America, Europe, and the former Soviet Union^49^. Major volcanic eruptions produced episodic sulfate aerosol perturbations superimposed on this anthropogenically influenced background. The subsequent decline in Arctic aerosol loading is consistent with reductions in anthropogenic sulfur emissions associated with air-quality regulations and broader socioeconomic changes^49,50^.

The close temporal correspondence between enhanced sulfate aerosol peaks and negative SAT anomalies, together with the causal volcanic-only forcing experiments from CMIP6 (hist-volc) shown in Fig. 1d–f, highlights the important role of volcanic aerosols in modulating short-term climate variability^21^. The divergence between simulations with and without volcanic forcing further demonstrates that volcanic eruptions temporarily suppressed Arctic warming. The estimated delay in Arctic warming reflects the cumulative effect of multiple transient cooling episodes rather than a sustained continuous cooling period. Each major eruption produced a cooling response lasting approximately 3–5 years, temporarily interrupting the warming trajectory before warming resumed, illustrating the important role of intermittent natural forcing in modulating long-term polar climate trends.

### Volcanic eruptions interrupted the polar warming observed from zonally averaged anomaly

**Fig. 2:**
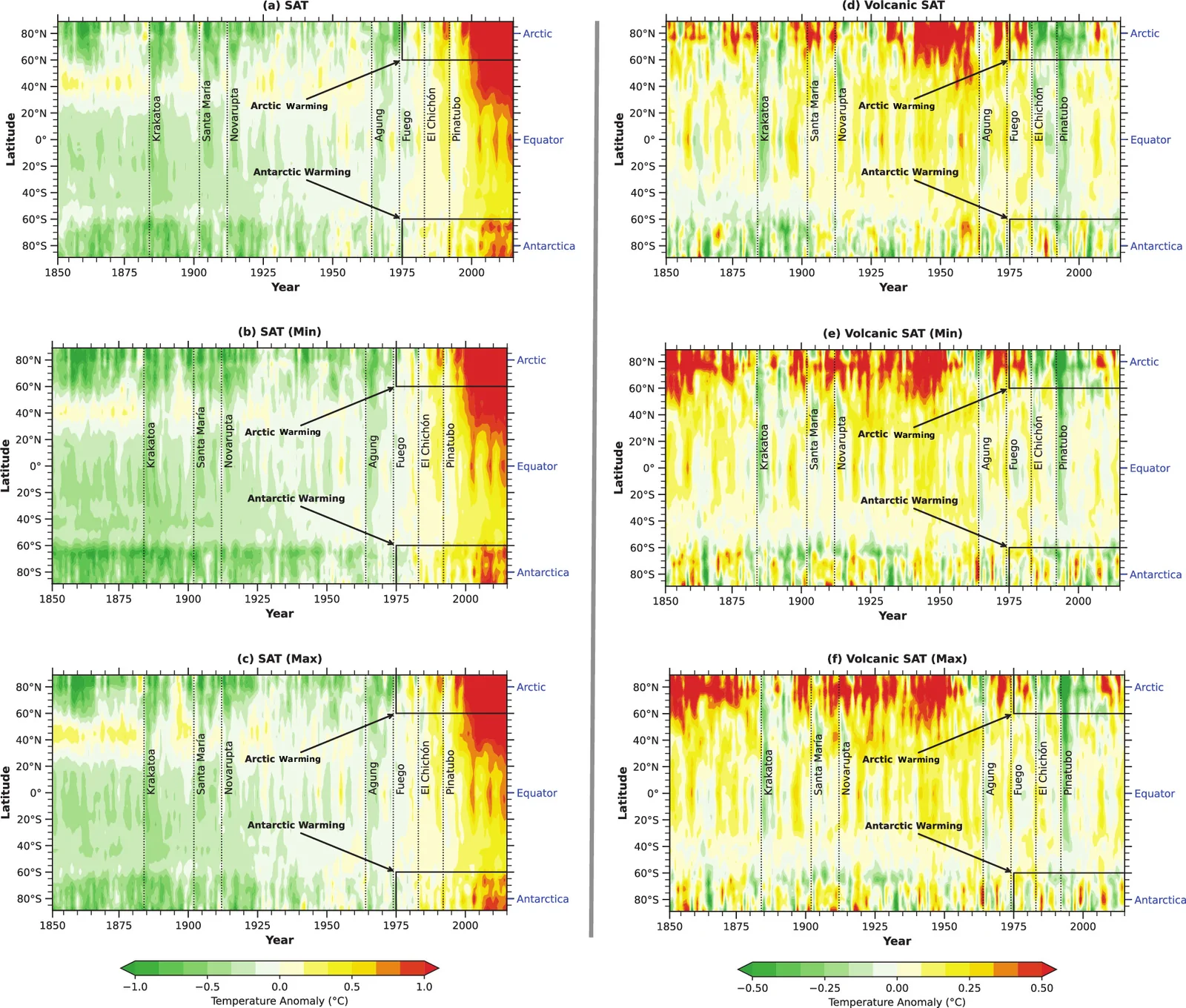
Zonally averaged temporal changes in SAT anomalies. **a**–**c** Zonally averaged annual anomalies of total SAT, (${T}_{\min }$), and (${T}_{\max }$) from all-forcing experiments. **d**–**f** Same as **a**–**c**, but for volcanic-only forcing experiments. Anomalies are computed relative to the mean over the volcanically influenced period (1964–1996). These panels highlight latitudinal differences in warming and cooling responses to total and volcanic-specific forcing.

**Fig. 3:**
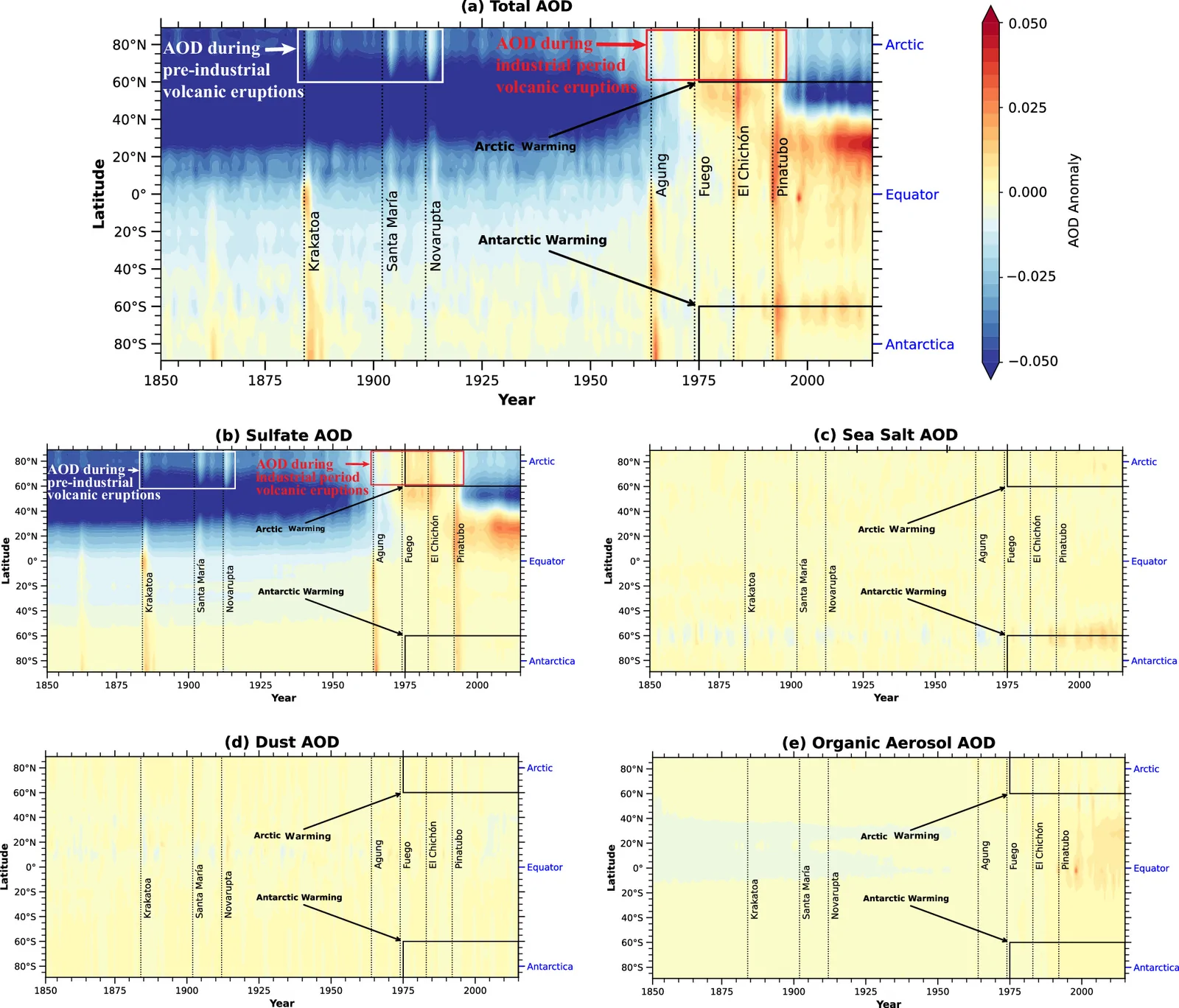
Zonally averaged annual anomalies in AOD. **a**–**e** Zonally averaged annual anomalies of total AOD, sulfate AOD, sea salt AOD, dust AOD, and organic AOD, respectively. Anomalies are determined in comparison to the mean over the volcanically influenced period (1964–1996). These panels illustrate the latitudinal distribution and temporal variability of different aerosol components, emphasizing the enhanced sulfate aerosol loading during major volcanic events.

To further elucidate the latitudinal and temporal modulation of polar warming by volcanic activity, the zonally averaged annual anomalies of total SAT, ${T}_{\min }$, and ${T}_{\max }$ derived from both all-forcing and volcanic-only CMIP6 multi-model ensemble simulations are shown in Fig. 2a–f. The zonally averaged total AOD and aerosol-component anomalies are shown in Fig. 3a–e. These fields represent the available CMIP6 aerosol output and are not interpreted as volcanic-only AOD. Volcanic perturbations are identified by the pronounced sulfate AOD enhancements coincident with major eruption events, while the volcanic contribution to the temperature response is independently evaluated using the hist-volc simulations.

In the all-forcing simulations (Fig. 2a–c), a robust latitudinal gradient in warming is evident in SAT, ${T}_{\min }$, and ${T}_{\max }$. The positive anomalies are not observed in the northern and southern high-latitude polar regions, particularly in the Arctic, after the mid-1970s. The polar warming has intensified post-2000 and is consistent with the Arctic and Antarctic warming. Among the SAT metrics, ${T}_{\min }$ exhibits greater anomaly magnitudes than ${T}_{\max }$ over polar regions (Fig. 2b vs. c), reflecting enhanced nocturnal warming and a narrowing annual temperature range. Critically, these warming patterns are interrupted by temporally distinct cooling effects centred around the latitudes of 60°–90°N, and 60°–90°S immediately following each major eruption (Fig. 2a–f). This indicates the transient radiative forcing exerted by volcanic aerosols. These volcanic aerosols increase planetary albedo by efficiently scattering incoming shortwave radiation and absorbing outgoing longwave radiation, thereby reducing net surface energy input^22^. Each major eruption during the volcanically influenced period (1964–1996) has suppressed Arctic total SAT, ${T}_{\min }$, and ${T}_{\max }$ anomalies for approximately 4–5 years (Fig. 2a–f).

Pronounced cooling anomalies occur at high latitudes following several major volcanic eruptions (Fig. 2a–f), illustrating the transient influence of volcanic radiative forcing on polar temperatures. Within the volcanically influenced period examined in detail here, the three major eruptions, Agung (1963), El Chichón (1982), and Pinatubo (1991) are associated with multi-year suppression of Arctic SAT, Tmin, and Tmax. These responses represent episodic interruptions of the Arctic warming trajectory, with warming resuming as the volcanic forcing diminishes. The Antarctic response is generally weaker and more spatially uniform, consistent with the moderating influences of Southern Ocean heat uptake, atmospheric circulation, and the thermal inertia of the Antarctic climate system^51,52^. The volcanic impact on the Arctic warming reflects the cumulative effect of multiple transient cooling episodes rather than a sustained continuous cooling phase. Each major eruption (Agung, El Chichón, and Pinatubo) produces a 4–5 year cooling period that temporarily suppresses warming, with warming resuming in between.

Moreover, climate variability modes, including the Arctic Oscillation (AO), North Atlantic Oscillation (NAO), and El Niño-Southern Oscillation (ENSO), can modulate surface temperature responses^53–55^. Major volcanic eruptions that coincide with strong phases of these modes may produce temperature anomalies reflecting both volcanic forcing and natural variability^54,56^. In addition, there is proxy evidence for an El Niño-like response to volcanic forcing, suggesting that eruptions can actively trigger ENSO-like conditions rather than simply overlapping with them^21,57^. While the use of multi-model means reduces the influence of internal variability (Fig. S3), we acknowledge that part of the observed signal could arise from these large-scale oscillations. Future work should further disentangle the comparative influence of volcanic forcing and natural climate variability.

It should also be noted that the temperature response to volcanic forcing can exhibit substantial seasonal dependence, particularly at high northern latitudes. Previous studies have reported contrasting winter and summer responses, with post-volcanic winter temperatures strongly influenced by dynamical changes involving the AO and NAO, which can locally produce winter warming despite the overall radiative cooling effect of volcanic aerosols^53–55^. The present analysis focuses on annual-mean SAT, Tmin, and Tmax because the primary objective is to quantify the multi-year modulation of the long-term polar warming trajectory. Consequently, season-specific responses are not separately diagnosed here. Future seasonally resolved analyzes would help distinguish the contributions of wintertime circulation changes and summertime radiative cooling to the annual-mean polar response.

The zonally averaged AOD anomalies and their major aerosol components are shown in Fig. 3a–e. Pronounced enhancements in total AOD occur in association with major volcanic eruptions, including Krakatoa (1883), Santa María (1902), Novarupta (1912), Agung (1963), El Chichón (1982), and Pinatubo (1991). These eruption-coincident enhancements are most clearly expressed in the sulfate AOD field (Fig. 3b), consistent with the formation and subsequent transport of stratospheric sulfate aerosols following major explosive eruptions. We emphasize that the CMIP6 AOD fields shown here are not volcanic-only diagnostics; therefore, the sulfate AOD field also contains contributions from non-volcanic sources. The pronounced sulfate enhancements coincident with major eruption events are used here to provide physical context for the volcanic temperature responses independently diagnosed from the hist-volc simulations.

Importantly, sulfate AOD anomalies are not confined to tropical latitudes but extend poleward into both hemispheres through Brewer–Dobson circulation and meridional transport processes, particularly during winter when cross-latitude mixing is enhanced^7,50,58–60^. The accumulation of sulfate aerosols over polar regions following major eruptions is evident as strong positive anomalies in Fig. 3b, confirming that volcanic aerosols are efficiently transported into polar stratospheric reservoirs. These aerosols enhance planetary shortwave reflectivity and produce radiative cooling through aerosol dimming effects^50^. The close temporal and zonal correspondence between enhanced sulfate AOD anomalies (Fig. 3b) and suppressed Arctic SAT, ${T}_{\min }$, and ${T}_{\max }$ anomalies (Fig. 2d–f) highlights the critical role of volcanic aerosol forcing in modulating polar climate variability and temporarily suppressing Arctic warming.

### Polar warming and cooling response with and without the volcanic eruptions and associated AOD changes

**Fig. 4:**
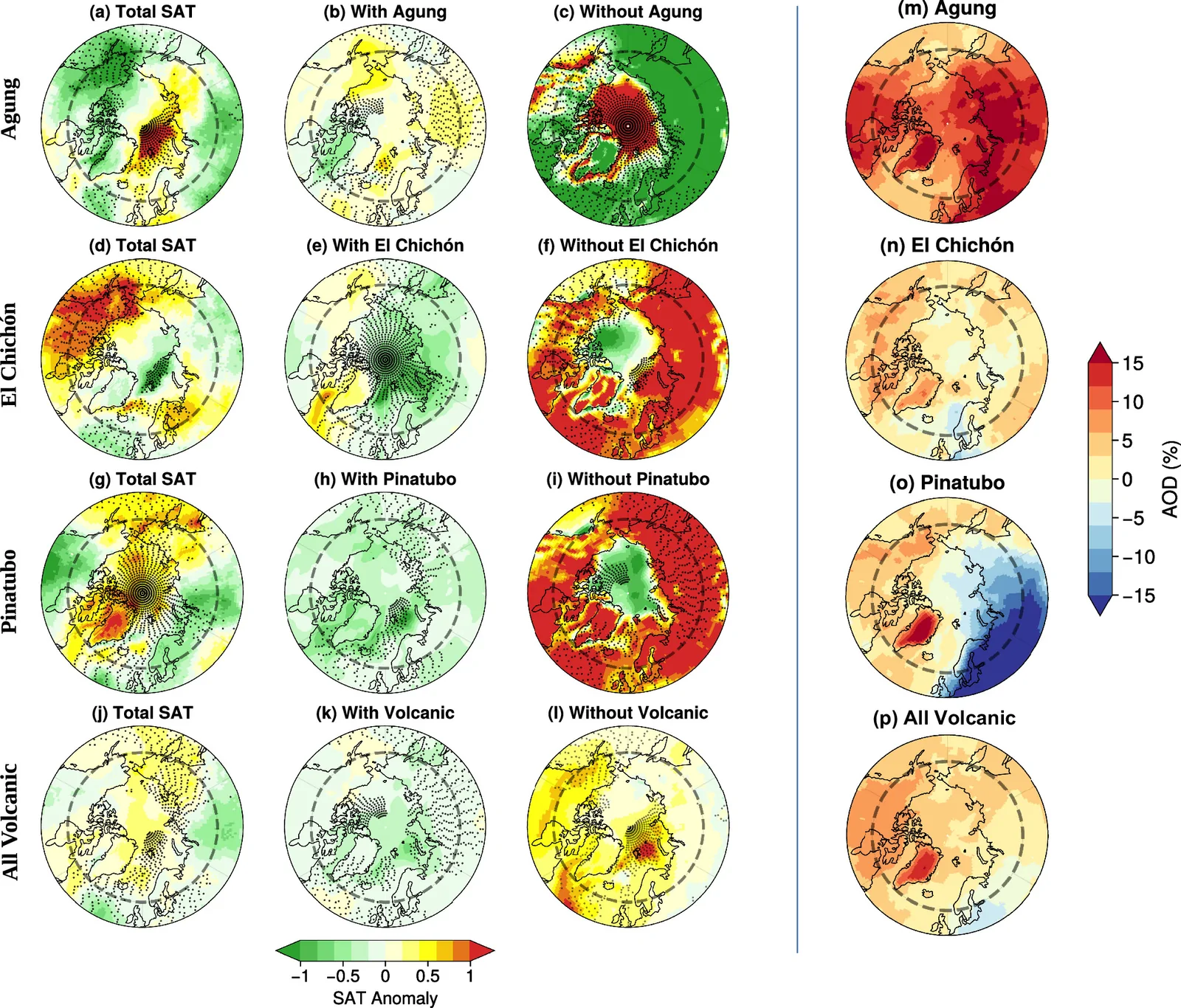
Spatial distribution of Arctic SAT anomalies with and without volcanic eruptions and associated AOD changes. Surface air temperature (SAT) anomalies associated with the Agung eruption, showing (**a**) total response, (**b**) simulations with volcanic forcing, and (**c**) simulations without volcanic forcing. Anomalies are calculated as the difference between the four-year post-eruption average (1964–1968) and the volcanically influenced baseline period (1964–1996). **d**–**f** Same as **a**–**c**, but for the El Chichón eruption (1983-1987). **g**–**i** Same as **a**–**c**, but for the Pinatubo eruption (1992–1996). Composite SAT anomalies representing the mean response across all three eruptions for (**j**) total response, (**k**) simulations with volcanic forcing, and (**l**) simulations without volcanic forcing. **m**–**o** Relative percentage changes in total aerosol optical depth (AOD) associated with the Agung, El Chichón, and Pinatubo eruptions, respectively, calculated as the difference between the four-year post-eruption average and the preceding four-year average, normalized by the pre-eruption baseline. **p** Composite relative AOD anomaly averaged across all three eruptions. Stippling indicates regions where SAT anomalies are statistically significant at the 95% confidence level based on a grid-point Student’s *t*-test. These panels highlight the spatial heterogeneity of Arctic temperature responses to volcanic forcing and the enhanced warming that emerges in the absence of volcanic aerosols.

**Fig. 5:**
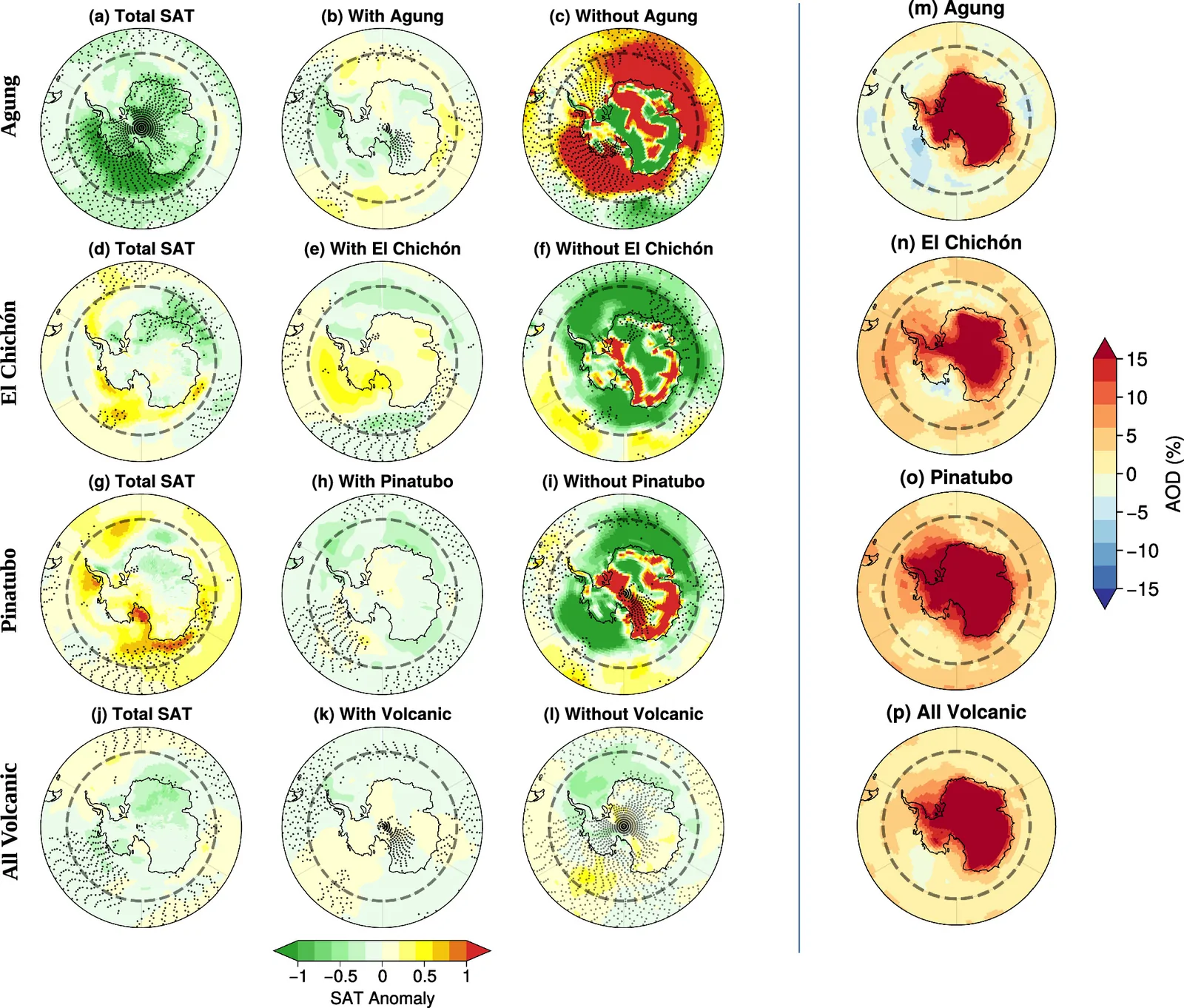
Spatial distribution of Antarctic SAT anomalies with and without volcanic eruptions and associated AOD changes. Surface air temperature (SAT) anomalies associated with the Agung eruption, showing (**a**) total response, (**b**) simulations with volcanic forcing, and (**c**) simulations without volcanic forcing. Anomalies are calculated as the difference between the four-year post-eruption average (1964–1968) and the volcanically influenced baseline period (1964–1996). **d**–**f** Same as **a**–**c**, but for the El Chichón eruption (1983–1987). **g**–**i** Same as **a**–**c**, but for the Pinatubo eruption (1992–1996). Composite SAT anomalies representing the mean response across all three eruptions for (**j**) total response, (**k**) simulations with volcanic forcing, and (**l**) simulations without volcanic forcing. **m**–**o** Relative percentage changes in total aerosol optical depth (AOD) associated with the Agung, El Chichón, and Pinatubo eruptions, respectively, calculated as the difference between the four-year post-eruption average and the preceding four-year average, normalized by the pre-eruption baseline. **p** Composite relative AOD anomaly averaged across all three eruptions. Stippling indicates regions where SAT anomalies are statistically significant at the 95% confidence level based on a grid-point Student’s *t*-test. These panels illustrate the comparatively weaker and more spatially heterogeneous Antarctic response to volcanic forcing, reflecting the moderating influence of the Southern Ocean and Antarctic atmospheric isolation.

To further investigate the role of volcanic eruptions on the spatial distribution of polar warming responses, we analyze the spatial anomalies of total SAT, ${T}_{\min }$, and ${T}_{\max }$, along with the relative percentage change in AOD, across the Arctic and Antarctic regions following three major volcanic eruptions—Agung (1963), El Chichón (1982), and Pinatubo (1991). This analysis is based on CMIP6 multi-model ensemble means, considering scenarios with and without volcanic forcing. The temperature anomalies are determined based on the difference from the 4-year post eruption mean and the volcanically influenced period (1964–1996) under both all-forcing and no-volcano scenarios (Figs. 4, 5 and S4–S8). The relative percentage changes in total AOD in the Arctic (Fig. 4m–p) for each eruption are calculated as the difference between the 4-year post eruption mean and the preceding 4-year mean, normalized by the pre-eruption baseline.

Over the Arctic, the spatial distribution of SAT anomalies associated with the Agung, El Chichón, and Pinatubo eruptions reveals pronounced regional heterogeneity in both temperature and aerosol responses (Fig. 4). The first three columns of Fig. 4 show the total SAT response, simulations with volcanic forcing, and simulations without volcanic forcing, respectively, for each eruption, while the final column presents the corresponding relative changes in AOD. Composite responses averaged across all eruptions are shown in the bottom row. For the Agung eruption, the total SAT response (Fig. 4a) shows cooling over Alaska, western Greenland, and the Canadian Archipelago, while warming occurs over northern Siberia and parts of the central Arctic. Simulations including volcanic forcing (Fig. 4b) indicate stronger cooling over the Canadian Archipelago and Arctic Ocean, with anomalies exceeding −0.5 °C. In contrast, the counterfactual simulations without volcanic forcing (Fig. 4c) exhibit widespread warming across Greenland and the central Arctic sea-ice region, with SAT anomalies exceeding +1 °C, while parts of Siberia show localized cooling. The Figs. S5a–c and S6a–c further show that, in the absence of the eruption, ${T}_{\min }$ warming is stronger than ${T}_{\max }$ over Greenland and the central Arctic, indicating enhanced sensitivity of minimum temperatures to volcanic suppression. The associated AOD changes during the Agung period (Fig. 4m) exceed 15% across Siberia, Greenland, and northern Canada. Although these elevated AOD values partly reflect volcanic forcing, they also include contributions from increasing anthropogenic sulfate emissions during the industrial period, particularly from Europe, North America, and the former Soviet Union.

The El Chichón eruption exhibits a spatial response distinct from Agung (Fig. 4d–f). The total SAT anomalies (Fig. 4d) show warming over the northern Canadian Arctic and cooling over northern Siberia and adjacent Arctic sea-ice regions. Simulations with volcanic forcing (Fig. 4e) produce widespread cooling over Siberian land and sea-ice regions, with SAT anomalies below −0.5 °C. In contrast, the simulations without volcanic forcing (Fig. 4f) show substantially stronger warming over Greenland, Arctic land regions, and open ocean areas, although parts of the central Arctic sea ice remain comparatively cooler. The corresponding ${T}_{\max }$ and ${T}_{\min }$ responses in Figs. S5d–f and S6d–f exhibit similar spatial structures, with stronger warming generally occurring in ${T}_{\min }$. Relative AOD anomalies during El Chichón (Fig. 4n) increase by more than 5% across most Arctic regions, indicating broad aerosol dispersion into the polar atmosphere.

The Pinatubo eruption also produces strong spatial contrasts in Arctic SAT anomalies (Fig. 4g–i). The total response (Fig. 4g) indicates warming over Greenland and parts of the central Arctic, while simulations including volcanic forcing (Fig. 4h) show widespread cooling over Greenland and sea-ice regions. In the absence of volcanic forcing (Fig. 4i), amplified warming emerges over Greenland, northern Canada, and Arctic land areas, with SAT anomalies exceeding +1 °C in several regions. The associated ${T}_{\max }$ and ${T}_{\min }$ patterns shown in Figs. S5g–l and S6g–l further indicate that minimum temperatures exhibit stronger warming anomalies than maximum temperatures in the no-volcano scenario. Relative AOD changes during Pinatubo (Fig. 4o) reveal increases over Greenland, Alaska, and northern Canada, while decreases occur over parts of the Siberian and European Arctic, indicating spatially varying aerosol transport and deposition patterns.

The composite Arctic response averaged across all eruptions (Fig. 4j–l) highlights the general influence of volcanic forcing on Arctic climate. The total SAT composite (Fig. 4j) indicates moderate cooling over large parts of the Arctic Ocean and Greenland. Simulations with volcanic forcing (Fig. 4k) show widespread cooling across the Arctic, whereas the counterfactual no-volcano simulations (Fig. 4l) exhibit substantially stronger warming over Greenland and Arctic land regions. The composite AOD anomaly (Fig. 4p) confirms enhanced aerosol loading over much of the Arctic during eruption periods, emphasizing the cumulative role of volcanic sulfate aerosols in suppressing Arctic warming.

A similar analysis for Antarctica is presented in Fig. 5. Compared to the Arctic, Antarctic SAT responses are generally weaker and more spatially heterogeneous, reflecting the stronger influence of Southern Ocean buffering, atmospheric isolation, and the thermal inertia of the Antarctic cryosphere. During the Agung eruption, the total SAT response (Fig. 5a) shows cooling across West Antarctica, the Ross Sea, and the Antarctic Peninsula, with weaker warming over parts of the Weddell Sea and East Antarctic coastal regions. Simulations with volcanic forcing (Fig. 5b) indicate enhanced cooling over West Antarctica and the Peninsula, while the no-volcano simulations (Fig. 5c) show widespread warming across oceanic regions and the Antarctic interior, with anomalies exceeding +1 °C. The Figs. S7a–c and S8a–c indicate stronger warming in ${T}_{\min }$ than in ${T}_{\max }$ over East Antarctica and adjacent oceanic regions in the absence of volcanic forcing. Relative AOD changes during Agung (Fig. 5m) exceed 15% across much of Antarctica, although anthropogenic influences are likely weaker than in the Arctic because of the relative isolation of Antarctic circulation.

The El Chichón eruption produces a distinct Antarctic response (Fig. 5d–f). The total SAT anomalies (Fig. 5d) show warming over the Southern Ocean and cooling near the Antarctic coast and Weddell Sea. With volcanic forcing (Fig. 5e), cooling intensifies along the northeastern Antarctic coast and Weddell Sea, whereas warming occurs over West Antarctica and the Ross Sea. In contrast, the no-volcano simulations (Fig. 5f) show stronger warming over coastal Antarctic regions and cooling over surrounding oceans. Similar spatial structures are evident in the corresponding ${T}_{\max }$ and ${T}_{\min }$ responses shown in Figs. S7d–f and S8d–f, with minimum temperatures generally exhibiting larger warming anomalies. Relative AOD anomalies during El Chichón (Fig. 5n) increase by 5–12% across much of Antarctica, particularly over the continental interior.

The Pinatubo eruption (Fig. 5g-i) produces cooling over East Antarctica and the Weddell Sea in the total SAT response, while slight warming occurs over West Antarctica and the Peninsula. Simulations including volcanic forcing (Fig. 5h) show widespread cooling over East Antarctica and the Weddell Sea, whereas the no-volcano simulations (Fig. 5i) exhibit stronger warming over the Antarctic Plateau and Peninsula, with anomalies exceeding +1 °C. The Figs. S7g–l and S8g–l further demonstrate that ${T}_{\min }$ anomalies are generally stronger than ${T}_{\max }$ anomalies in the absence of volcanic forcing. Relative AOD changes during Pinatubo (Fig. 5o) exceed 15% over central Antarctica, the Peninsula, and the Weddell Sea, consistent with efficient transport of volcanic aerosols into the polar stratosphere.

The composite Antarctic response across all eruptions (Fig. 5j–l) shows weaker and more spatially diffuse cooling compared to the Arctic. Simulations with volcanic forcing (Fig. 5k) exhibit modest cooling over much of Antarctica, whereas simulations without volcanic forcing (Fig. 5l) show stronger warming over the Antarctic interior and Peninsula. The composite AOD anomaly (Fig. 5p) confirms enhanced aerosol loading over Antarctica during eruption periods, although the associated SAT response remains weaker than in the Arctic because of stronger oceanic buffering and atmospheric isolation. Overall, the spatial analyzes demonstrate that volcanic eruptions produce regionally heterogeneous effects on SAT, ${T}_{\min }$, and ${T}_{\max }$ across both polar regions, with the Arctic exhibiting substantially stronger sensitivity to volcanic aerosol forcing than Antarctica.

**Fig. 6:**
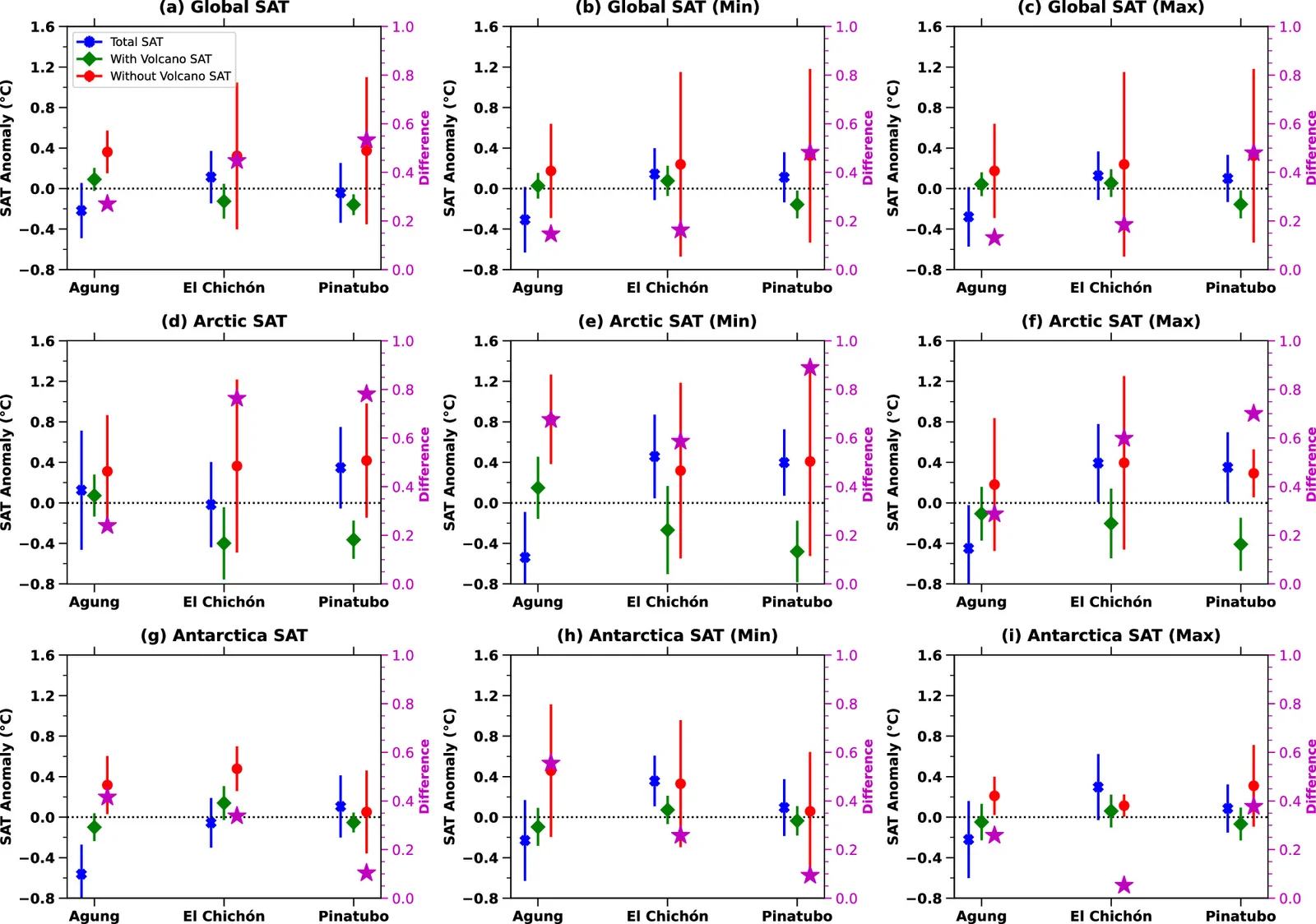
Average SAT anomalies and one standard deviation, including minimum (${T}_{\min }$) and maximum (${T}_{\max }$) SAT, with and without volcanic eruptions over the Global, Arctic, and Antarctic regions. **a**–**c** Mean anomalies and associated one standard deviation bars for SAT, ${T}_{\min }$, and ${T}_{\max }$ with and without the Agung, El Chichón, and Pinatubo eruptions, based on CMIP6 multi-model ensemble means for the Global average. **d**–**f** Same as **a**–**c**, but for the Arctic region. **g**–**i** Same as **a**–**c**, but for Antarctica. Anomalies are determined based on the difference from the four-year post-eruption averages (1964–1968 for Agung, 1983–1987 for El Chichón, and 1992–1996 for Pinatubo) and the long-term volcanically influenced period (1964–1996). The values represent spatial averages over the global, Arctic, and Antarctica region. The mean solid circle and one standard deviation vertical line for total SAT, with volcanic eruption SAT, and without volcanic eruption SAT are presented as blue, green, and red colors, respectively. The maroon-colored star associated with the second *Y*-axis shows the difference between the surface air temperature without volcanic eruption and with volcanic eruption.

Figure 6a–i summarizes the spatial average of SAT, ${T}_{\max }$, and ${T}_{\min }$ anomalies and one standard deviation ranges for global, Arctic, and Antarctic regions following Agung, El Chichón, and Pinatubo eruptions. Anomalies are determined in comparison to the volcanically influenced period (1964–1996), with post-eruption periods defined as 1964–1968 (Agung), 1983–1987 (El Chichón), and 1992–1996 (Pinatubo). Based on CMIP6 ensemble means, vertical bars represent inter-model spread. Globally, volcanic forcing results in cooling of SAT, with the Pinatubo eruption showing the largest effect: a mean SAT anomaly of −0.15 °C compared to +0.37 °C without volcanic forcing—a net difference of 0.52 °C (Fig. 6a). Similar global SAT differences are observed for Agung (0.27 °C) and El Chichón (0.44 °C). ${T}_{\min }$ and ${T}_{\max }$ follow the same pattern as SAT, indicating no significant divergence in global maximum and minimum temperature responses to volcanic forcing (Fig. 6b, c).

The Arctic shows a more amplified response (Fig. 6d–f). For instance, after Pinatubo, SAT with volcanic forcing declines by −0.36 °C, whereas the no-volcano scenario indicates warming of +0.41 °C, yielding a net suppression of 0.8 °C (Fig. 6d). For El Chichón, SAT drops by −0.39 °C with volcanic forcing, while Agung shows a small warming (+0.07 °C), in contrast to stronger warming (+0.31 to +0.36 °C) without volcanic input. Arctic ${T}_{\min }$ anomalies exceed −0.3 °C during El Chichón and Pinatubo, while ${T}_{\max }$ remains closer to −0.3 °C (Fig. 6e, f). High inter-model variability is evident, with standard deviations of 0.25–0.65 °C. Antarctica shows a more muted response (Fig. 6g–i), with SAT anomalies from −0.09 °C (Agung) to −0.05 °C (Pinatubo) under volcanic forcing, and weak warming (<0.3 °C) without it. Net differences remain small (0.15–0.3 °C). ${T}_{\min }$ and ${T}_{\max }$ anomalies are also closely aligned, and standard deviations are lower (0.1–0.3 °C), reflecting the region’s thermal inertia and isolated atmospheric circulation. Maroon stars in each panel indicate the net difference in SAT between simulations with and without volcanic forcing, plotted against a secondary *y*-axis. These values highlight the regional variability in volcanic impacts, with the Arctic experiencing the greatest suppression of warming (up to 0.9 °C), followed by the global mean (0.3 °C), and Antarctica (0.2 °C). These findings reinforce the relevance of including volcanic forcing in climate analyzes, particularly for high-latitude regions where the response is more sensitive and pronounced. The response to the Agung eruption differs from the other events, with a weaker cooling signal and, in some regions, a slight warming tendency. This likely reflects the relatively smaller magnitude of the eruption, differences in injection latitude and aerosol distribution, as well as the influence of internal climate variability. Previous studies have shown that such factors can lead to regionally heterogeneous and even opposing temperature responses following volcanic eruptions. Despite this variability, the overall multi-event response remains characterized by net cooling, particularly for the larger eruptions such as El Chichón and Pinatubo.

### Implications of historical volcanic cooling for future geoengineering of polar Warming

**Fig. 7:**
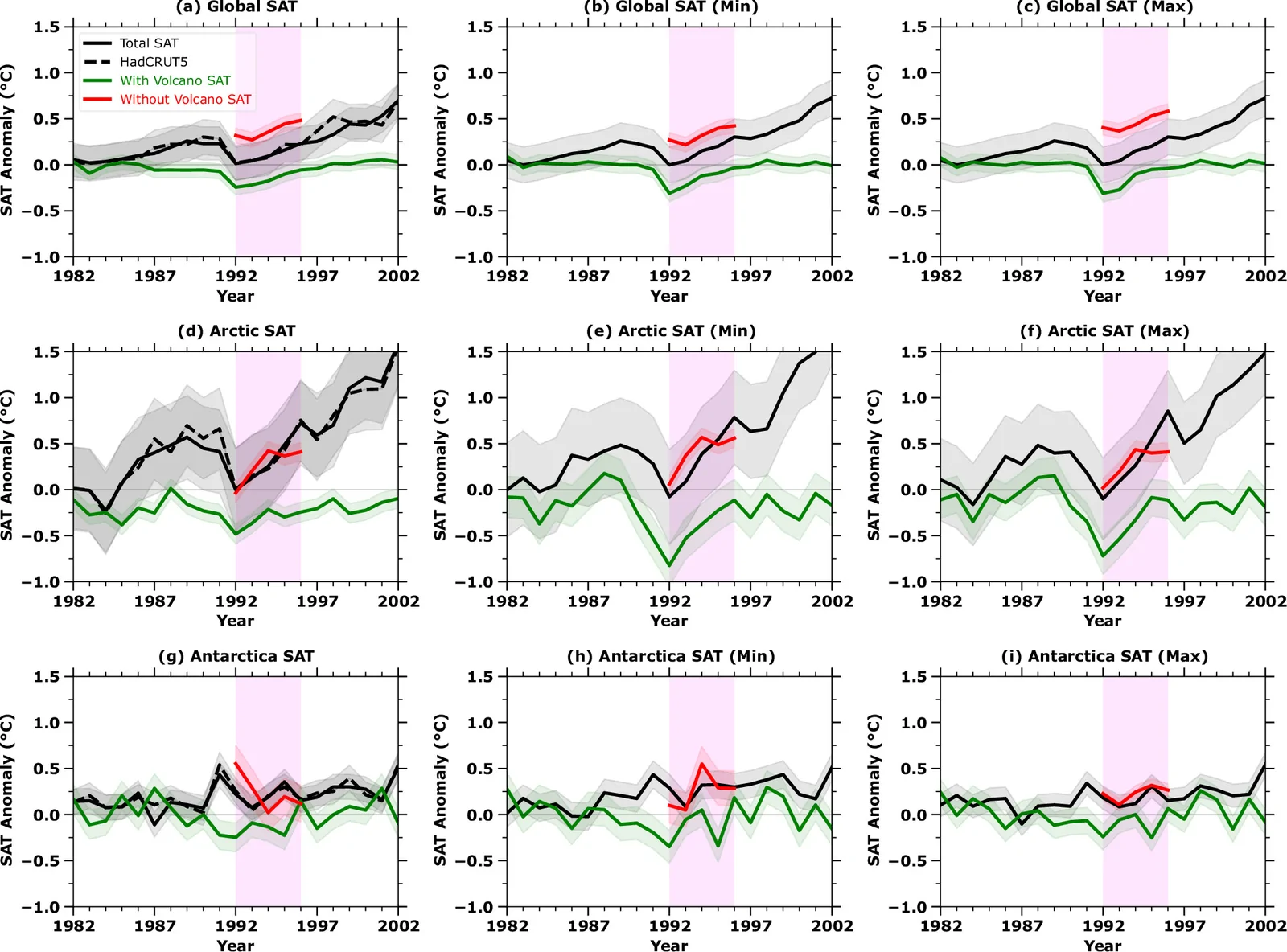
Temporal variation of SAT anomalies, including minimum (${T}_{\min }$) and maximum (${T}_{\max }$) SAT, with and without volcanic eruptions over the Global, Arctic, and Antarctic regions for the Pinatubo eruption. **a**–**c** Time series of anomalies in SAT, ${T}_{\min }$, and ${T}_{\max }$ for Global averages, estimated from the CMIP6 multi-model mean, with and without volcanic forcing. **d**–**f** Same as **a**–**c**, but for the Arctic region. **g**–**i** Same as **a**–**c**, but for Antarctica. Anomalies are determined in comparison to the volcanically influenced period (1964–1996). The figure includes SAT time series from the CMIP6 multi-model mean (black solid line), with volcanic forcing (green line), without volcanic forcing (red line), and from HadCRUT5 SAT observations with (black dotted line). These comparisons illustrate the transient cooling impact of the Pinatubo eruption on temperature evolution across the Global, Arctic, and Antarctica.

To assess the relevance of historical volcanic events for potential geoengineering strategies, we analyze the transient cooling impacts of the 1991 Mount Pinatubo eruption on global and polar total SAT, ${T}_{\min }$, and ${T}_{\max }$ using CMIP6 multi-model-mean simulations (Fig. 7a–i). Pinatubo represents the largest late-20th-century stratospheric sulfate perturbation during a period of accelerating Arctic warming, and is widely regarded as the most robust natural analogue for proposed solar radiation management (SRM) approaches, particularly stratospheric aerosol injection (SAI)^21,61,62^. Figure 7, therefore, provides a grounded benchmark for evaluating how sulfate-driven radiative forcing modifies polar warming.

Globally (Fig. 7a–c), Pinatubo induces a rapid cooling of approximately 0.1–0.2 °C that persists for about 4–5 years. The divergence between simulations with and without volcanic forcing (green versus red curves) highlights the degree to which stratospheric aerosols suppress SAT rise. The modeled evolution closely follows HadCRUT5 observations (black dotted line), reinforcing confidence that CMIP6 closely captures the magnitude and timing of sulfate-induced radiative perturbations. Even short-lived radiative perturbations from volcanic aerosols can influence cryospheric processes by altering the timing of melt onset and surface albedo feedbacks, particularly in the Arctic where small energy imbalances can trigger amplified sea-ice responses^4,54,62^. Similar global cooling magnitudes have been simulated in Geoengineering Model Intercomparison Project Stratospheric Aerosol Injection (GeoMIP SAI) experiments under idealized sulfate injection scenarios^63,64^, suggesting that volcanic analogues provide a physically consistent reference for SRM modeling studies.

The Arctic response (Fig. 7d–f) is substantially amplified. Following Pinatubo, Arctic SAT declines by nearly 0.6 °C, with ${T}_{\min }$ reductions approaching 0.8 °C. This pronounced sensitivity underscores the strong coupling between radiative forcing, boundary-layer stability, and sea-ice feedbacks in high latitudes. The sharper response in ${T}_{\min }$ relative to ${T}_{\max }$ indicates that sulfate aerosols disproportionately affect the radiative cooling and surface energy balance, a feature not commonly emphasized in SRM assessments but potentially critical for understanding impacts on extreme cold events, sea-ice thermodynamics, and cryospheric processes^42,44,65^. Geoengineering simulations have likewise reported enhanced Arctic cooling under sulfate injection scenarios^66–68^, reinforcing the interpretation that high latitudes are especially responsive to stratospheric aerosol perturbations.

In contrast, Antarctica (Fig. 7g–i) exhibits a much weaker and shorter-lived response, with SAT reductions limited to 0.1–0.2 °C. This hemispheric asymmetry reflects differences in stratospheric circulation, seasonal aerosol transport, and the thermal inertia of the Southern Ocean^7^. Comparable asymmetries have been identified in SRM modeling studies, where regional outcomes depend strongly on injection latitude and atmospheric dynamics^69^. The weaker Antarctic response suggests that globally uniform SAI may not symmetrically suppress polar warming, raising important questions about regional impact. Further, the relatively weak temperature response over the Antarctic interior is not solely attributable to oceanic buffering. In addition to the moderating influence of the Southern Ocean, the Antarctic Plateau is characterized by high elevation, persistent surface-based temperature inversions, and atmospheric isolation associated with the circumpolar vortex. These factors limit both vertical mixing and horizontal transport of heat anomalies, reducing the sensitivity of the continental interior to short-term radiative perturbations such as volcanic forcing^42,65^.

While Fig. 7 demonstrates the technical feasibility of aerosol-induced cooling, it also highlights the intrinsic limitations of volcanic analogues. The cooling effect decays within several years as aerosols are removed from the stratosphere, indicating that sustained injection would be required to maintain temperature suppression^70,71^. Moreover, stratospheric sulfate loading is associated with dynamical and chemical adjustments, including stratospheric heating and ozone perturbations^47,72,73^. Although our analysis focuses on surface temperature, previous studies show that SAI may weaken the global hydrological cycle and alter precipitation patterns^67,74^. These side effects are not visible in Fig. 7 but are critical when extrapolating short-term volcanic cooling to sustained geoengineering deployment.

Another key insight from Fig. 7 is the rapid rebound of warming once volcanic forcing diminishes, a phenomenon analogous to the “termination shock” described in SRM simulations^75^. The transient suppression of the Arctic warming following Pinatubo suggests that immediate cessation of sustained aerosol injection could lead to accelerated warming, particularly in sensitive high-latitude systems. This reinforces concerns raised in recent assessments that SRM introduces long-term dependency and governance challenges^76,77^.

Overall, Fig. 7 provides anchored evidence that stratospheric sulfate aerosols can temporarily suppress global and Arctic warming, with Arctic cooling approaching 0.8 °C in minimum temperatures. These results are consistent with SAI modeling studies and GeoMIP experiments^63^, yet they also underscore the transient nature, spatial heterogeneity, and potential side effects of aerosol-based interventions. Historical volcanic eruptions thus serve as indispensable natural experiments for evaluating the climatic response to radiative perturbations, while simultaneously illustrating the profound complexities and risks associated with translating volcanic analogues into deliberate geoengineering strategies aimed at mitigating polar warming.

## Discussion

This study systematically investigates the influence of major volcanic eruptions on total SAT, minimum temperature ${T}_{\min }$, and maximum temperature ${T}_{\max }$, with a particular focus on polar regions, using CMIP6 multi-model simulations. Previous studies have established that large volcanic eruptions induce short-term global cooling through stratospheric sulfate aerosol formation^19,78,79^. Through zonally averaged diagnostics, spatial anomaly maps, and time series analyzes, we provide robust evidence that stratospheric volcanic aerosols have exerted substantial short-term cooling effects globally and in the polar regions^4,21^. Our results are consistent with these earlier findings but extend them by providing a detailed assessment of polar responses and the differential behavior of ${T}_{\min }$ and ${T}_{\max }$.

Analysis of zonally averaged SAT anomalies (Fig. 2a–f) reveals that the Arctic and to a lesser extent, Antarctica experienced a notable delay in the onset of warming following major eruptions such as Agung, El Chichón, and Pinatubo. Similar temporary offsets of greenhouse gas-driven warming by volcanic forcing have been reported in previous studies^80,81^. This delayed warming is most evident in the suppression of ${T}_{\min }$, which consistently shows stronger cooling than ${T}_{\max }$ in the Arctic (Fig. 2). Volcanic-only forcing simulations isolate this transient cooling effect, confirming that it arises from stratospheric aerosols rather than other anthropogenic or natural influences.

The transient volcanic cooling identified here also provides context for the slowdown in global surface warming during the early 21st century. This slowdown resulted from a combination of internal climate variability and external forcings, with moderate volcanic eruptions contributing through enhanced stratospheric aerosol loading^81^. Our results support this mechanism and further indicate that volcanic suppression of warming can be particularly pronounced in the Arctic. Thus, volcanic forcing can temporarily modulate, but does not reverse, the long-term anthropogenic warming trend.

These latitudinally stratified cooling patterns are aligned closely to peaks in zonally averaged AOD, particularly sulfate AOD, as shown in Fig. 2. Sulfate aerosols released in large quantities during volcanic eruptions are well known to dominate shortwave radiative forcing^19,79^. Elevated concentrations extend across the polar latitudes and align with the observed cooling responses. Spatial anomaly maps (Figs. 4 and 5) further highlight regional differences in post-eruption cooling. In the Arctic, the strongest cooling is centered over central Siberia, Greenland, and the Arctic Ocean, while in Antarctica, cooling is more heterogeneous, with stronger anomalies over East Antarctica and the Antarctic Peninsula. These spatial patterns are consistent with previous studies showing that aerosol transport pathways and atmospheric circulation play a key role in shaping volcanic climate impacts^82,83^. They reflect the complex interplay between aerosol transport, regional radiative balance, and atmospheric circulation.

Decomposition of SAT into ${T}_{\min }$ and ${T}_{\max }$ (Figs. S3–S6) reveals that ${T}_{\min }$ is disproportionately sensitive to volcanic forcing, often more so than ${T}_{\max }$. This suggests that stratospheric aerosol loading enhances nighttime radiative cooling, likely through increased atmospheric stability and reduced longwave radiation, thereby amplifying ${T}_{\min }$ suppression relative to daytime temperatures. Similar diurnal asymmetries have been reported in studies of aerosol forcing and volcanic impacts on diurnal temperature range^42,43^. The time series analyzes centered on the 1991 Mount Pinatubo eruption (Fig. 7a–i) offer a more detailed temporal view of these effects. Globally, the eruption produced an immediate cooling of approximately 0.1–0.2 °C that persisted for about 3 to 4 years, consistent with earlier observational and modeling studies^19,84^. This response was stronger in ${T}_{\min }$ than ${T}_{\max }$, consistent with nocturnal radiative effects. In the Arctic, cooling reached up to 0.6 °C in SAT and nearly 0.8 °C in ${T}_{\min }$, significantly dampening the warming trajectory during this period. This short-term suppression is clearly visible in both model outputs and HadCRUT5 observations, manifesting as a strong V-shaped dip in the early 1990s. Simulations without volcanic forcing show a steeper Arctic warming trend, further emphasizing the cooling influence of volcanic aerosols^21^. In contrast, the Antarctic response was more muted, with cooling limited to about 0.1–0.2 °C, primarily in ${T}_{\min }$.

The Pinatubo eruption, as the largest eruption during a period of rapid Arctic warming, provides a crucial natural experiment for evaluating the feasibility of climate intervention approaches such as SRM. The injection of sulfur dioxide into the stratosphere led to the formation of sulfate aerosols that reflected incoming shortwave radiation, producing a measurable global cooling of up to 0.5 °C over 2 years^19^. As such, Pinatubo has frequently been used as an analog for SAI^61,62^. Climate model intercomparison studies, such as GeoMIP^63^, have demonstrated that sulfate aerosol injection can offset global warming, including in high latitudes. Our results provide additional support for this, showing that volcanic aerosols can temporarily suppress Arctic warming. However, they also highlight key limitations: the cooling effects are short-lived, spatially uneven, and influenced by complex dynamical responses. Previous studies have further emphasized potential side effects of SAI, including impacts on precipitation and atmospheric chemistry^47,74^.

In conclusion, our study yields several key insights: (1) volcanic eruptions have transiently suppressed polar warming, especially in the Arctic, consistent with previous studies, by inducing strong but short-lived cooling through sulfate aerosol loading; this suppression occurs as a series of episodic cooling events lasting 4–5 years following each major eruption, which temporarily interrupt the warming trajectory and cumulatively affected the Arctic warming. (2) ${T}_{\min }$ exhibits a more pronounced response than ${T}_{\max }$, underscoring the importance of analyzing these temperature metrics separately in attribution and mitigation studies; and (3) spatial variability in SAT and AOD anomalies highlights the greater sensitivity of the Arctic compared to Antarctica and points to the role of aerosol transport, circulation dynamics, and regional feedback processes. Understanding the historical modulation of polar climate by volcanic aerosols is thus not only critical for attributing observed climate change, but also for rigorously assessing the feasibility and ethics of future climate engineering efforts aimed at mitigating polar warming.

Future work should focus on improving the representation of volcanic aerosol processes and their interactions with clouds, boundary-layer dynamics, and sea-ice feedbacks in climate models. In particular, better constraints on high-latitude aerosol transport, Arctic moisture feedbacks, and the differential response of minimum and maximum temperatures would enhance our understanding of polar climate sensitivity to radiative forcing. Additional studies exploring regionally targeted aerosol perturbations and their dynamical consequences would also help refine projections of polar climate responses.

Beyond physical climate implications, these findings have broader social and political relevance. The transient and spatially heterogeneous cooling effects associated with volcanic aerosols highlight the challenges of applying similar mechanisms through geoengineering approaches such as SAI^61^. Potential risks include uneven regional climate impacts, uncertainties in hydrological responses, and the possibility of warming following termination of interventions. These factors underscore the need for careful international governance, ethical considerations, and continued emphasis on emissions mitigation alongside any exploration of climate intervention strategies.

## Methods

### Climate models used in this study

This study employs multi-model ensemble simulations from the Coupled Model Intercomparison Project Phase 6 (CMIP6)^39^ to investigate the climate response to three major volcanic eruptions of the 20th century: Agung (1963), El Chichón (1982), and Pinatubo (1991). To isolate the climatic influence of volcanic aerosols on polar warming, we analyze outputs from complementary CMIP6 experiment categories, including historical simulations^39^, volcanic-only simulations (hist-volc)^85^, and the Decadal Climate Prediction Project counterfactual experiment dcppC-hindcast-noVolc^86^. The historical simulations include all major anthropogenic and natural forcings, including greenhouse gases, anthropogenic aerosols, volcanic aerosols, solar variability, and land-use changes, spanning the period 1850–2014. In contrast, hist-volc simulations isolate volcanic forcing by prescribing volcanic aerosol perturbations while excluding or holding other forcings fixed, depending on model configuration. The dcppC-hindcast-noVolc experiment consists of initialized decadal hindcasts that exclude volcanic forcing, thereby providing a counterfactual climate state without major volcanic eruptions^86^. This experimental framework enables comparison between forced and counterfactual climate conditions and allows assessment of the contribution of volcanic aerosols relative to internally varying climate states. The analyzed CMIP6 simulations cover the standard historical period used for the IPCC Sixth Assessment Report and build upon earlier coordinated CMIP experiments. Model outputs include historical, hist-volc, and dcppC-hindcast-noVolc simulations (Tables 1 and S1). To maintain methodological consistency, the same ensemble member (r1i1p1f1) was used across all models, as volcanic-only simulations are most consistently available for this realization. Ensemble means were calculated only using models that provided all required simulations. Surface air temperature anomalies associated with each eruption were computed using 4-year post eruption averages: 1964–1968 for Agung, 1983–1987 for El Chichón, and 1992–1996 for Pinatubo. A 4-year averaging period was selected to capture both the immediate and residual climatic response to volcanic forcing while minimizing the influence of interannual variability.

**Table 1:** Summary of CMIP6 experiments used in this study, including experiment names, forcings, and their roles in the analysis

Experiment name	Forcings included	Purpose in study	Label used
Historical	GHG + anthropogenic aerosols + volcanic + solar + land-use	Represents total external forcing over the historical period	All-forcing
Hist-volc	Volcanic aerosols only (other forcings fixed or absent)	Isolates the climatic impact of volcanic forcing	Volcanic-only
dcppC-hindcast-noVolc	Initialized simulations excluding volcanic forcing	Counterfactual climate simulations without volcanic aerosols	Without volcanic forcing

Temperature anomalies are calculated relative to the volcanically influenced reference period (1964–1996), which encompasses the major eruptions of Agung (1963), El Chichón (1982), and Pinatubo (1991). This reference period is intentionally used to provide a common framework for comparing the temperature responses associated with these eruption events. Consequently, negative anomalies during earlier portions of the record indicate temperatures below the 1964–1996 mean and should not be interpreted as evidence for the absence of anthropogenic warming during those periods. The choice of reference period defines the anomaly zero point but does not define the onset of Arctic or anthropogenic warming.

While the use of a volcanically influenced baseline can produce negative anomalies during periods of low aerosol loading, these anomalies remain physically meaningful because they reflect reduced background aerosol concentrations, including lower anthropogenic sulfate loading during earlier decades. This contrast enhances the detectability of eruption-driven signals and facilitates the interpretation of volcanic perturbations in both temperature and aerosol fields. Although volcanic-only AOD components would provide a more direct representation of eruption signals, such outputs are not consistently available in CMIP6 simulations; therefore, total AOD and its dominant sulfate component are used to identify volcanic aerosol signatures.

The analysis focuses on three spatial domains: the global mean, the Arctic (60°–90°N), and the Antarctic (60°–90°S). The Arctic domain is defined as latitudes north of 60°N, consistent with commonly used high-latitude diagnostics in the Arctic warming studies and CMIP analyzes^4,7,46^. This definition represents a climatological high-latitude domain rather than a geopolitical Arctic definition and captures the region of strongest Arctic warming. Three temperature metrics were analyzed: mean surface air temperature (SAT), minimum temperature (${T}_{\min }$), and maximum temperature (${T}_{\max }$). SAT corresponds to the near-surface (2 m) air temperature variable (tas) from CMIP6, while ${T}_{\min }$ and ${T}_{\max }$ correspond to annual means of daily minimum (tasmin) and daily maximum (tasmax) near-surface air temperatures, respectively. All anomalies represent deviations from the 1964–1996 baseline period. All model outputs were interpolated to a uniform 1° × 1° grid using bilinear interpolation with Climate Data Operators (CDO)^87^ to ensure inter-model comparability.

The volcanic forcing in CMIP6 models is prescribed using externally derived stratospheric AOD datasets rather than through fully interactive aerosol microphysics. These forcing datasets are typically provided as zonal-mean or latitude-height-time distributions that represent the radiative effects of sulfate aerosols formed from volcanic SO_2_ emissions^39,85^. Most CMIP6 models, therefore, impose volcanic aerosol radiative forcing through prescribed AOD fields, which ensures consistency across models but may underestimate peak aerosol loading and associated radiative forcing. It is important to distinguish between stratospheric volcanic aerosols and tropospheric anthropogenic aerosols. Major volcanic eruptions inject sulfur dioxide (SO_2_) into the stratosphere^88^, where it oxidizes to form sulfate aerosols with atmospheric lifetimes of approximately 1–3 years^18,22,23^. These aerosols produce globally distributed radiative forcing. In contrast, anthropogenic tropospheric aerosols are shorter-lived and regionally concentrated. The hist-volc and dcppC-hindcast-noVolc experiments, therefore, represent prescribed stratospheric sulfate aerosol perturbations rather than tropospheric aerosol emissions.

To facilitate interpretation, the mapping between CMIP6 experiments, included forcings, and analysis labels used in this study is summarized in Table 1.

Model performance was evaluated using Taylor diagrams (Figs. S1a, b and S2), which compare simulated SAT and AOD against observational datasets over the global, Arctic, and Antarctic domains. HadCRUT5^40^ was used for SAT validation, while MERRA-2 reanalysis^41^ was used for AOD evaluation. The Taylor diagram for SAT (Fig. S2) shows appreciable inter-model differences in correlation, normalized standard deviation, and centered root-mean-square error. HadGEM3-GC31-LL and CMCC-CM2-SR5 show relatively closer agreement with HadCRUT5, whereas MIROC6 and CanESM5 exhibit larger departures in variability and centered error; ACCESS-ESM1-5 and MRI-ESM2-0 show intermediate performance. These differences indicate that individual models represent observed temperature variability with different levels of skill. Nevertheless, the multi-model mean reduces the influence of individual model-specific variability and provides a more robust estimate of the common forced response^39^. The observed inter-model spread likely reflects differences in the representation of atmospheric circulation, aerosol forcing, sea-ice processes, and related climate feedbacks, although these individual contributions are not separately diagnosed in the present analysis. The CMIP6 MMM reproduces the broad observed SAT variability, while volcanic AOD peaks are generally underestimated relative to MERRA-2, particularly over the Arctic and globally (Fig. S3a–d).

The validation period (1980–2014) corresponds to the modern observational era with extensive satellite and ground-based coverage. The CMIP6 MMM reproduces observed SAT variability well, while volcanic AOD peaks are generally underestimated relative to MERRA-2, particularly over the Arctic and globally (Fig. S3a–d). AOD is a dimensionless column-integrated measure of aerosol extinction of solar radiation, representing the cumulative scattering and absorption of radiation by aerosols throughout the atmospheric column^48^. Higher AOD values indicate greater aerosol loading and stronger radiative attenuation.

Observed SAT anomalies were derived from area-weighted HadCRUT5 monthly data relative to the 1964–1996 baseline period. The median observational record was used throughout the analysis. Relative changes in AOD associated with each eruption were additionally evaluated to provide physical context for the modeled temperature responses and to illustrate the evolution of aerosol loading during volcanic events. We emphasize that the relationship between AOD and SAT presented here is not based on statistical correlation, but rather reflects causally forced responses from controlled CMIP6 simulations in which volcanic forcing is explicitly prescribed. The AOD fields are therefore shown to illustrate aerosol loading and its temporal coincidence with temperature responses.

Statistical significance of SAT anomalies in the spatial maps was evaluated using a grid-point one-sample Student’s *t*-test against a null hypothesis of zero anomaly, following commonly used approaches in climate variability analysis^89,90^. For each eruption period, SAT anomalies were tested independently at each latitude-longitude grid point across the temporal dimension. Grid points with *p*-values less than 0.05 were considered statistically significant at the 95% confidence level, and only statistically significant anomalies were retained in the masked significance maps. All analyzes were performed using Python and CDO, providing a robust framework for quantifying volcanic impacts on regional and global climate variability.

## Supplementary information

Supplementary Information

## Data Availability

All the CMIP6 model datasets are available at https://esgf-data.dkrz.de/search/CMIP6-dkrz/. Observational data from HadCRUT5 is available at https://crudata.uea.ac.uk/cru/data/temperature/. MERRA2 data is available at https://gmao.gsfc.nasa.gov/reanalysis/merra-2/.

## Code availability

All the data analysis and the maps generated in the manuscript and supplement are done by using publicly available Python version 3.5.0.
