# Supplementary material for: Major volcanic eruptions episodically disrupted the continuity of polar warming through aerosol forcing
Source: NPJ Clean Air. 2026 Nov 1;2(1):67. doi: 10.1038/s44407-026-00111-3 (PMC13724269; doi:10.1038/s44407-026-00111-3)
Supplement: Supplementary file 1 [file 44407_2026_111_MOESM1_ESM.pdf]

Major volcanic eruptions episodically disrupted the continuity of Arctic warming through aerosol forcing: Supplement

Basudev Swain[1,*], Scott Osprey[2,*], Roy G. Grainger[1,*], Rui Song[1], and Marco Vontas[3]

[1]NCEO; Atmospheric, Oceanic and Planetary Physics, University of Oxford, UK
[2]Department of Physics, Atmospheric, Oceanic and Planetary Physics, University of Oxford, U.K
[3]Institute of Environmental Physics (IUP), University of Bremen, Germany
[*]Corresponding, Basudev Swain: basudev.swain@physics.ox.ac.uk, Scott Osprey: scott.osprey@physics.ox.ac.uk; Roy G. Grainger: don.grainger@physics.ox.ac.uk

Supplement Data

Model Name	Institution	Volcanic Forcing Experiments	Resolution
ACCESS-ESM1-5	CSIRO-BOM (Australia)	`historical`, `hist-volcano`	$\sim$1.875° × 1.25°
CanESM5	CCCma (Canada)	`historical`, `hist-volcano`	$\sim$2.8° × 2.8°
CMCC-CM2-SR5	CMCC (Italy)	`historical`, `hist-volcano`	$\sim$1.0° × 1.0°
HadGEM3-GC31-LL	Met Office Hadley Centre (UK)	`historical`, `hist-volcano`	$\sim$1.875° × 1.25°
MIROC6	MIROC (Japan)	`historical`, `hist-volcano`	$\sim$1.4° × 1.4°
MRI-ESM2-0	MRI (Japan)	`historical`, `hist-volcano`	$\sim$1.125° × 1.125°

Table S1. The CMIP6 Models with Surface Air Temperature (SAT) Simulations Including Volcanic Forcing

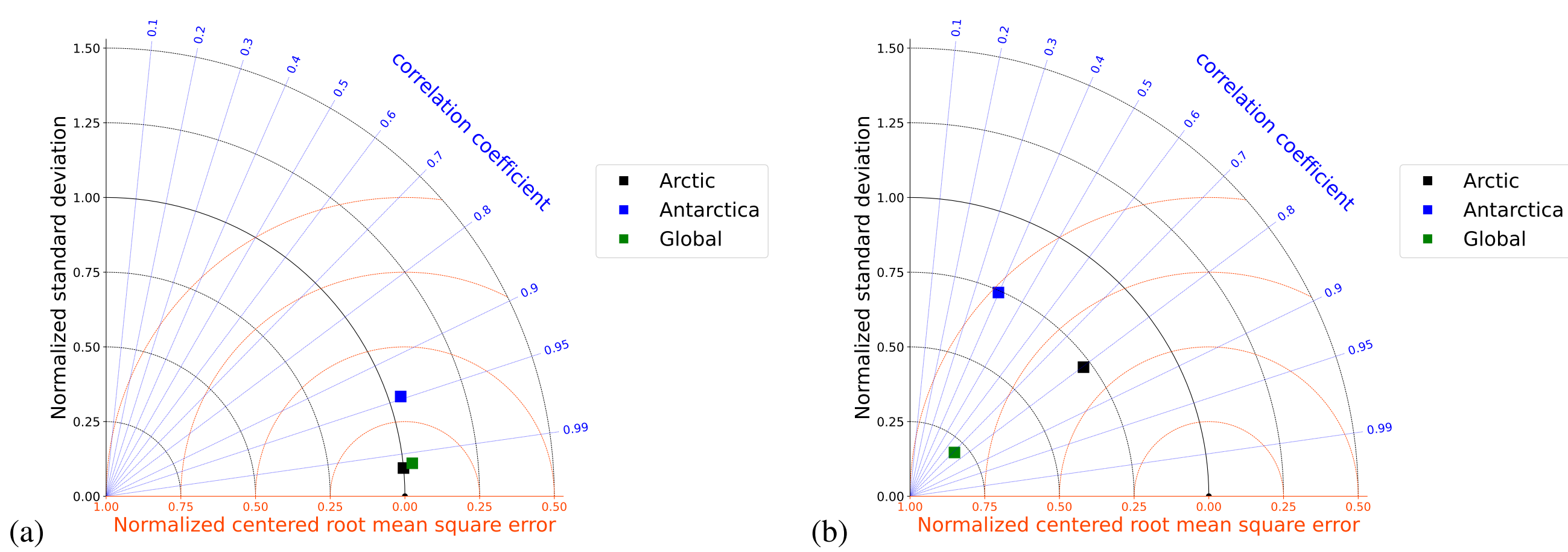

Figure S1. Taylor diagrams evaluating multi-model means against observational datasets over 1980–2015 for the Arctic, Antarctica, and Globe. (a) Taylor diagram comparing mean surface air temperature (SAT) from the CMIP6 multi-model mean with HadCRUT5 observations. (b) Taylor diagram comparing total aerosol optical depth (AOD) from the CMIP6 multi-model mean with MERRA-2 reanalysis data.

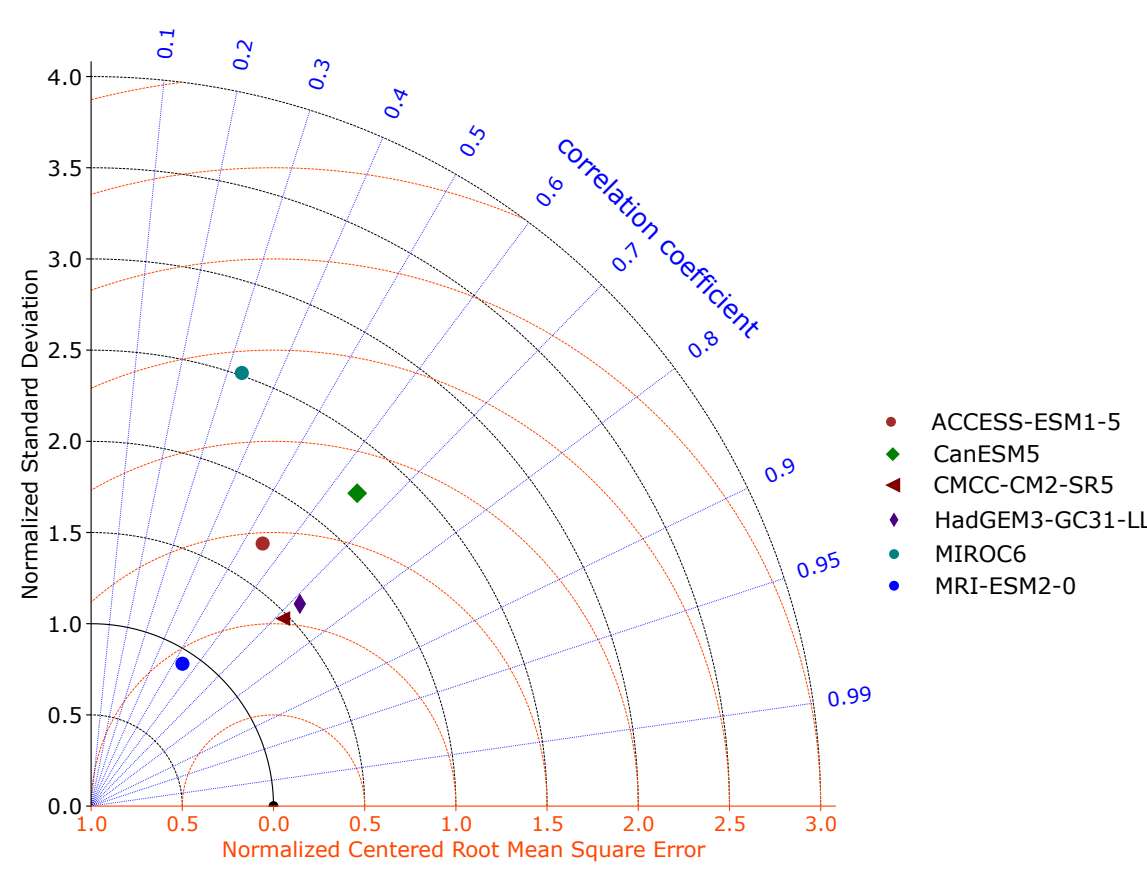

Figure S2. Taylor diagram of CMIP6 model performance relative to HadCRUT5 observations. Comparison of surface air temperature (SAT) simulated by the individual CMIP6 models with HadCRUT5 observations. Model performance is evaluated using the correlation coefficient, normalized standard deviation, and normalized centered root-mean-square error. The observational reference is represented by the reference point with a normalized standard deviation of 1 and zero centered error. Models located closer to the reference point indicate better overall agreement with the observed SAT variability. The diagram highlights the inter-model spread and differences in the representation of observed temperature variability among the CMIP6 models.

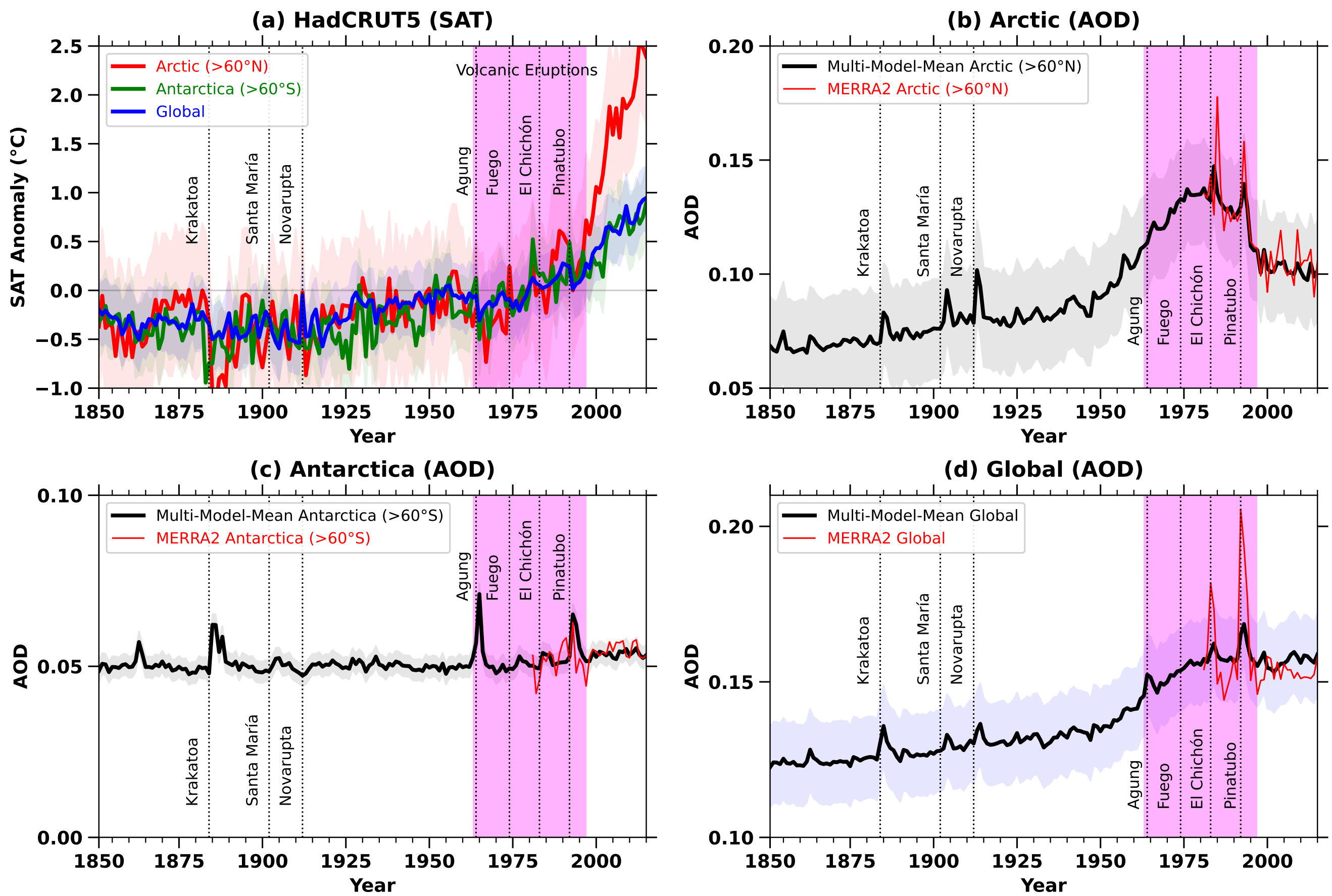

Figure S3. Temporal variations in surface air temperature (SAT) anomalies and total aerosol optical depth (AOD). (a) Annual anomalies of total SAT from HadCRUT5 observations over the Global (blue), Arctic (red), and Antarctic (green) regions. (b–d) Temporal evolution of total AOD over the Arctic, Antarctica, and Global regions, derived from the CMIP6 multi-model ensemble mean and MERRA-2 reanalysis datasets. These panels highlight the inverse relationship between AOD peaks associated with major volcanic eruptions and corresponding reductions in SAT, illustrating the cooling effect of volcanic aerosols on surface temperatures.

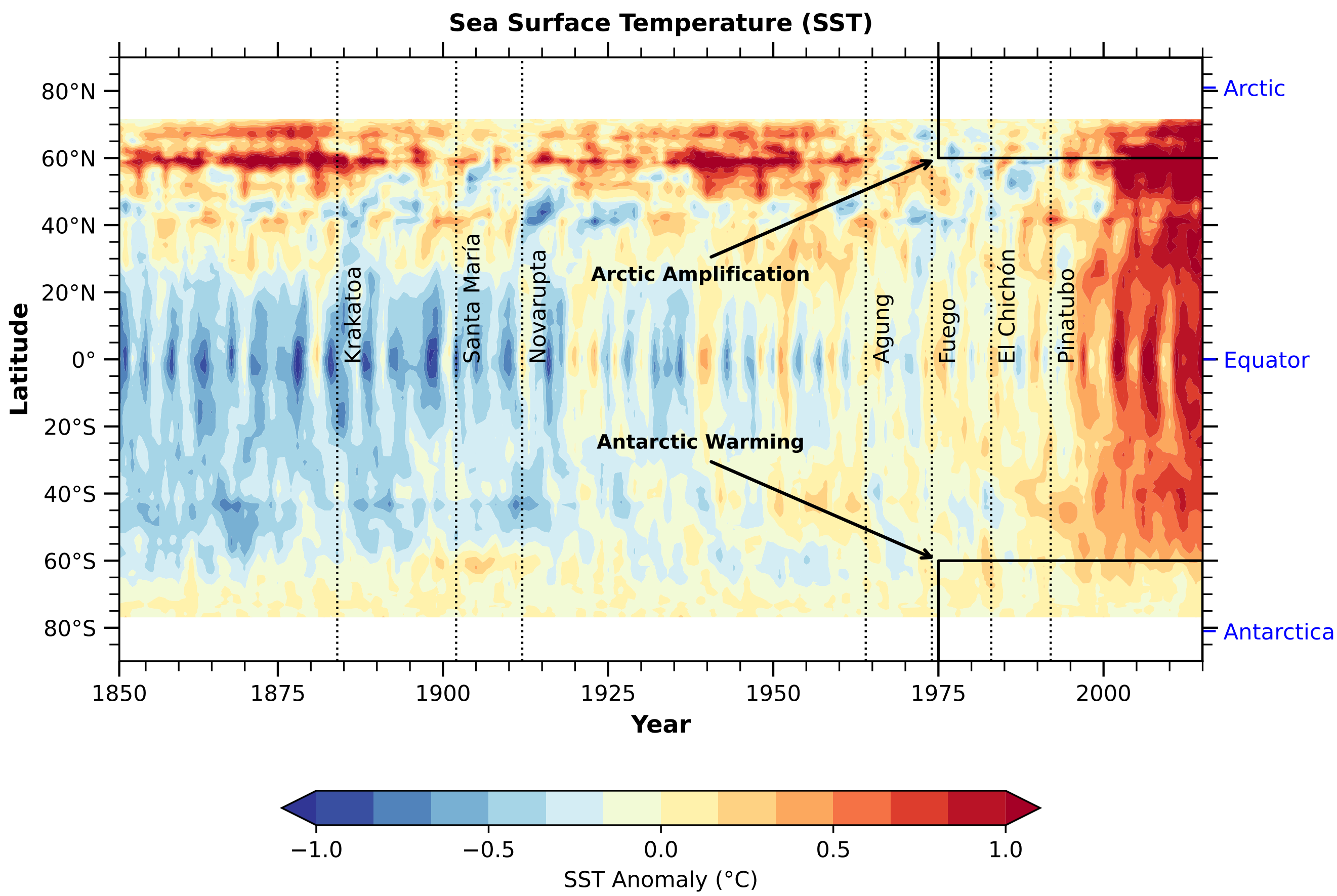

Figure S4. Zonally averaged temporal changes in Sea Surface Temperature (SST) anomalies. Zonally averaged annual anomalies of total SST from all-forcing experiments. Anomalies are computed relative to the mean over the volcanic reference period (1964–1996).

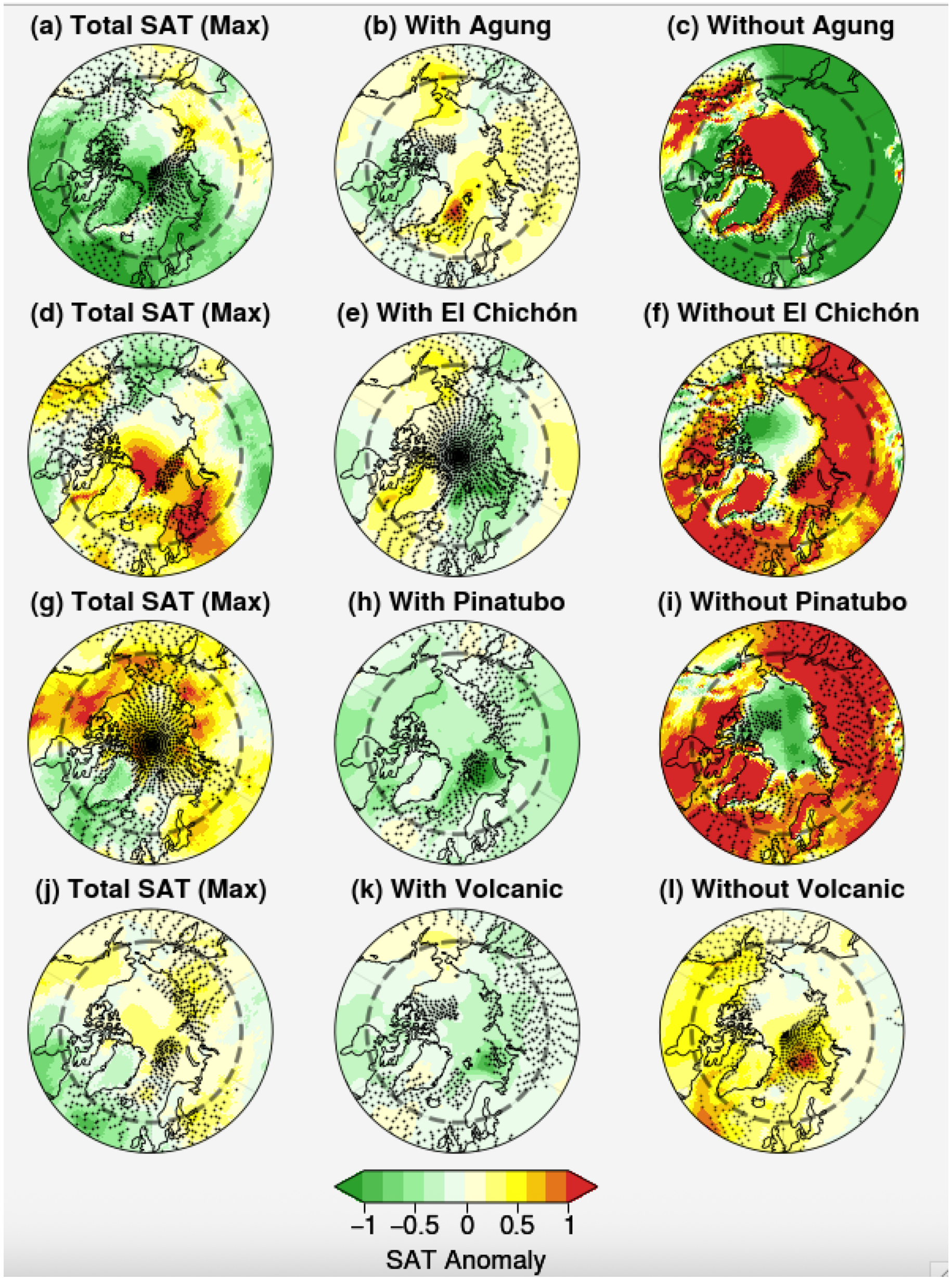

Figure S5. Spatial distribution of Arctic T_{max} anomalies with and without volcanic eruptions. (a–c) Maximum surface air temperature (T_{max}) anomalies associated with the Agung eruption, showing (a) total response, (b) simulations with volcanic forcing, and (c) simulations without volcanic forcing. Anomalies are calculated as the difference between the four-year post-eruption average (1964–1968) and the volcanically influenced baseline period (1964–1996). (d–f) Same as (a–c), but for the El Chichón eruption (1983–1987). (g–i) Same as (a–c), but for the Pinatubo eruption (1992–1996). (j–l) Composite T_{max} anomalies representing the mean response across all three eruptions for (j) total response, (k) simulations with volcanic forcing, and (l) simulations without volcanic forcing. Stippling indicates regions where anomalies are statistically significant at the 95% confidence level based on a grid-point Student's *t*-test. These panels highlight the spatial heterogeneity of volcanic impacts on Arctic maximum temperatures and the enhanced warming that emerges in the absence of volcanic forcing.

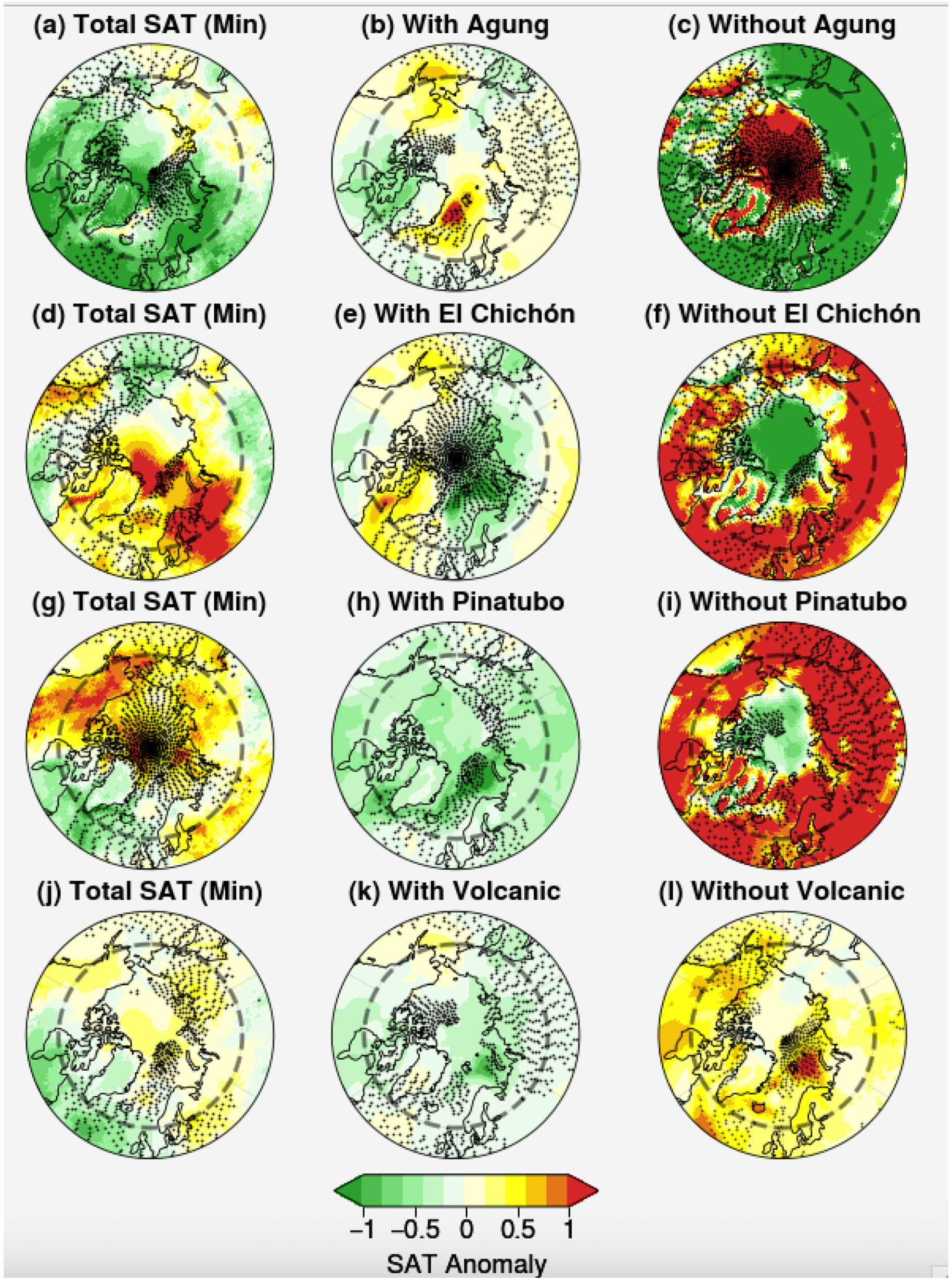

Figure S6. Spatial distribution of Arctic $T_{\min}$ anomalies with and without volcanic eruptions. (a–c) Minimum surface air temperature ($T_{\min}$) anomalies associated with the Agung eruption, showing (a) total response, (b) simulations with volcanic forcing, and (c) simulations without volcanic forcing. Anomalies are calculated as the difference between the four-year post-eruption average (1964–1968) and the volcanically influenced baseline period (1964–1996). (d–f) Same as (a–c), but for the El Chichón eruption (1983–1987). (g–i) Same as (a–c), but for the Pinatubo eruption (1992–1996). (j–l) Composite $T_{\min}$ anomalies representing the mean response across all three eruptions for (j) total response, (k) simulations with volcanic forcing, and (l) simulations without volcanic forcing. Stippling indicates regions where anomalies are statistically significant at the 95% confidence level based on a grid-point Student's t-test. These panels highlight the spatial heterogeneity of volcanic impacts on Arctic maximum temperatures and the enhanced warming that emerges in the absence of volcanic forcing.

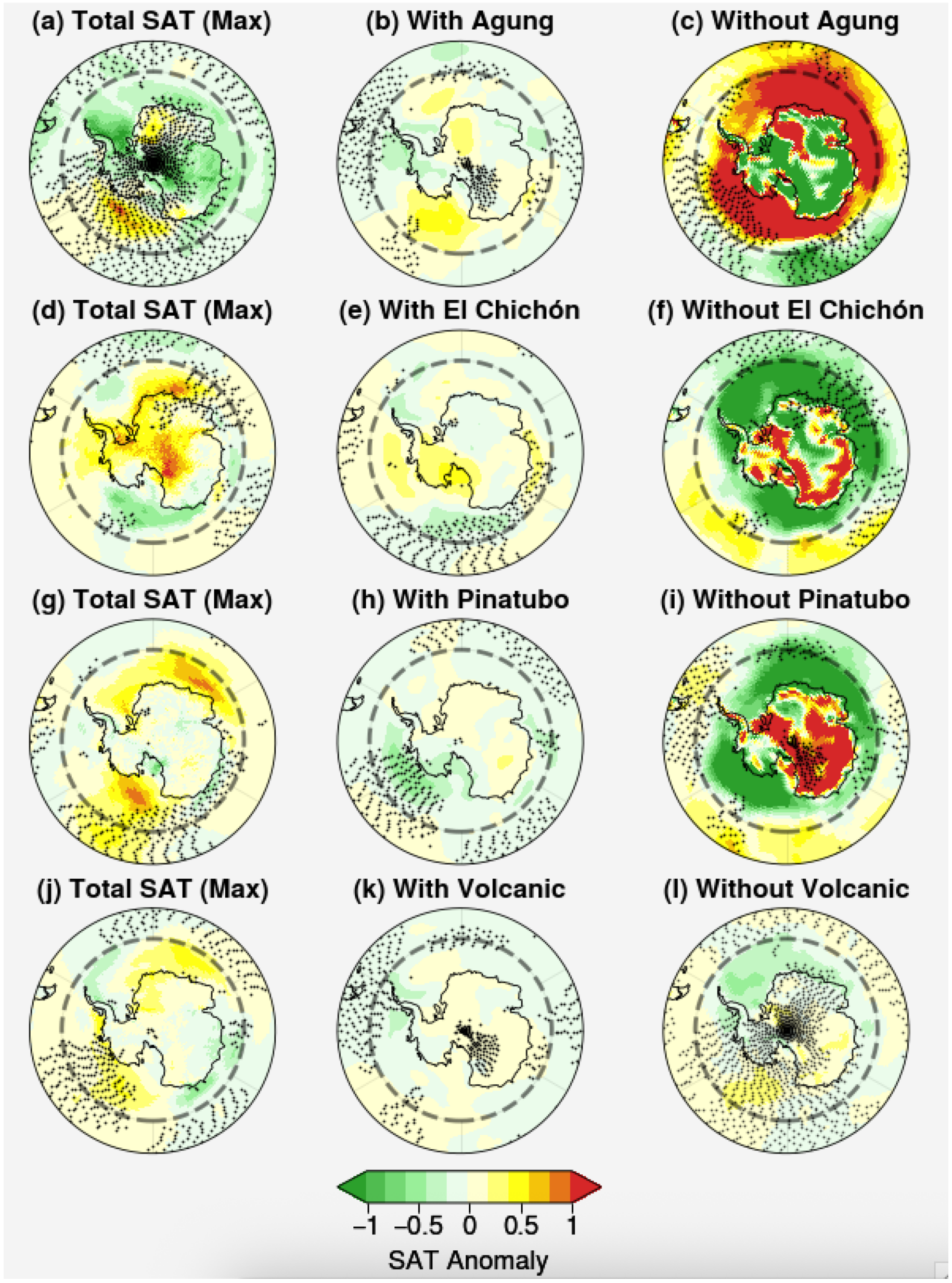

Figure S7. **Spatial distribution of Antarctic T_{max} anomalies with and without volcanic eruptions.** (a–c) Maximum surface air temperature (T_{max}) anomalies associated with the Agung eruption, showing (a) total response, (b) simulations with volcanic forcing, and (c) simulations without volcanic forcing. Anomalies are calculated as the difference between the four-year post-eruption average (1964–1968) and the volcanically influenced baseline period (1964–1996). (d–f) Same as (a–c), but for the El Chichón eruption (1983–1987). (g–i) Same as (a–c), but for the Pinatubo eruption (1992–1996). (j–l) Composite T_{max} anomalies representing the mean response across all three eruptions for (j) total response, (k) simulations with volcanic forcing, and (l) simulations without volcanic forcing. Stippling indicates regions where anomalies are statistically significant at the 95% confidence level based on a grid-point Student's *t*-test. These panels highlight the spatial heterogeneity of volcanic impacts on Arctic maximum temperatures and the enhanced warming that emerges in the absence of volcanic forcing.

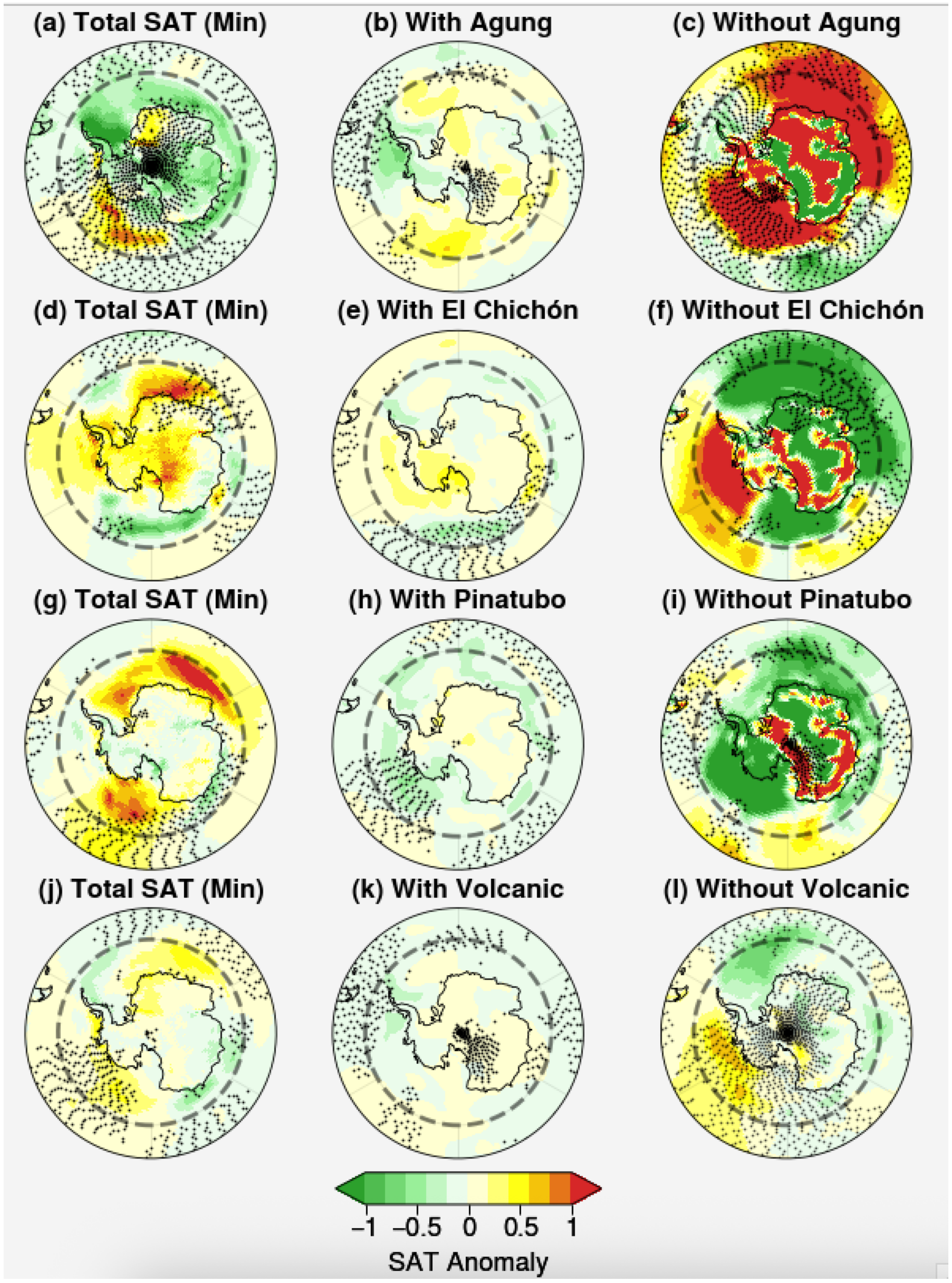

Figure S8. Spatial distribution of Antarctic T_{min} anomalies with and without volcanic eruptions. (a–c) Minimum surface air temperature (T_{min}) anomalies associated with the Agung eruption, showing (a) total response, (b) simulations with volcanic forcing, and (c) simulations without volcanic forcing. Anomalies are calculated as the difference between the four-year post-eruption average (1964–1968) and the volcanically influenced baseline period (1964–1996). (d–f) Same as (a–c), but for the El Chichón eruption (1983–1987). (g–i) Same as (a–c), but for the Pinatubo eruption (1992–1996). (j–l) Composite T_{min} anomalies representing the mean response across all three eruptions for (j) total response, (k) simulations with volcanic forcing, and (l) simulations without volcanic forcing. Stippling indicates regions where anomalies are statistically significant at the 95% confidence level based on a grid-point Student's *t*-test. These panels highlight the spatial heterogeneity of volcanic impacts on Arctic maximum temperatures and the enhanced warming that emerges in the absence of volcanic forcing.
